# Multi-omics landscape to decrypt the distinct flavonoid biosynthesis of *Scutellaria baicalensis* across multiple tissues

**DOI:** 10.1093/hr/uhad258

**Published:** 2023-11-13

**Authors:** Dandan Guo, Zhenyu Zhu, Zhe Wang, Fei Feng, Qi Cao, Zhewei Xia, Xinlei Jia, Diya Lv, Ting Han, Xiaofei Chen

**Affiliations:** Pharmaceutical Analysis and Testing center, School of Pharmacy, Naval Medical University (Second Military Medical University), Shanghai, 200433, China; Shanghai Key Laboratory for Pharmaceutical Metabolite Research, Shanghai, 200433, China; Pharmaceutical Analysis and Testing center, School of Pharmacy, Naval Medical University (Second Military Medical University), Shanghai, 200433, China; Shanghai Key Laboratory for Pharmaceutical Metabolite Research, Shanghai, 200433, China; Department of Physiology, College of Basic Medical Sciences, Naval Medical University (Second Military Medical University), Shanghai, 200433, China; Pharmaceutical Analysis and Testing center, School of Pharmacy, Naval Medical University (Second Military Medical University), Shanghai, 200433, China; Shanghai Key Laboratory for Pharmaceutical Metabolite Research, Shanghai, 200433, China; Pharmaceutical Analysis and Testing center, School of Pharmacy, Naval Medical University (Second Military Medical University), Shanghai, 200433, China; Shanghai Key Laboratory for Pharmaceutical Metabolite Research, Shanghai, 200433, China; Pharmaceutical Analysis and Testing center, School of Pharmacy, Naval Medical University (Second Military Medical University), Shanghai, 200433, China; Shanghai Key Laboratory for Pharmaceutical Metabolite Research, Shanghai, 200433, China; Pharmaceutical Analysis and Testing center, School of Pharmacy, Naval Medical University (Second Military Medical University), Shanghai, 200433, China; Pharmaceutical Analysis and Testing center, School of Pharmacy, Naval Medical University (Second Military Medical University), Shanghai, 200433, China; Shanghai Key Laboratory for Pharmaceutical Metabolite Research, Shanghai, 200433, China; Pharmaceutical Analysis and Testing center, School of Pharmacy, Naval Medical University (Second Military Medical University), Shanghai, 200433, China; Pharmaceutical Analysis and Testing center, School of Pharmacy, Naval Medical University (Second Military Medical University), Shanghai, 200433, China; Shanghai Key Laboratory for Pharmaceutical Metabolite Research, Shanghai, 200433, China

## Abstract

*Scutellaria baicalensis* Georgi, also known as huang-qin in traditional Chinese medicine, is a widely used herbal remedy due to its anticancer, antivirus, and hepatoprotective properties. The *S. baicalensis* genome was sequenced many years ago; by contrast, the proteome as the executer of most biological processes of *S. baicalensis* in the aerial parts, as well as the secondary structure of the roots (xylem, phloem, and periderm), is far less comprehensively characterized. Here we attempt to depict the molecular landscape of the non-model plant *S. baicalensis* through a multi-omics approach, with the goal of constructing a highly informative and valuable reference dataset. Furthermore, we provide an in-depth characterization dissection to explain the two distinct flavonoid biosynthesis pathways that exist in the aerial parts and root, at the protein and phosphorylated protein levels. Our study provides detailed spatial proteomic and phosphoproteomic information in the context of secondary structures, with implications for the molecular profiling of secondary metabolite biosynthesis in non-model medicinal plants.

## Introduction

*Scutellaria baicalensis* Georgi, also referred to as huang-qin, is a species of medicinal plant that is widely cultivated in China, East Asia and some European countries [1–3]. As a traditional Chinese medicine (TCM), its dried root has been used for anticancer [4–8], hepatoprotection [9, 10], antibacterial and antiviral activity for thousands of years [11]. The pharmacological properties of *S. baicalensis* have been largely attributed to its extensive accumulation of flavonoids, including baicalein, scutellarein, norwogonin, wogonin, and their glycosides (baicalin, scutellarin, norwogonoside, and wogonoside) [12–14]. Both the aerial parts and the roots of *S. baicalensis* produce flavones. Scutellarein and scutellarin are the primary flavones found in the aerial organs, while baicalein, baicalin, wogonin, wogonoside, norwogonin, and norwogonoside are the most abundant flavones present in the roots [14]. This situation might provide an intriguing avenue for enhancing the production of bioactive compounds in plants or synthesizing them in novel hosts.

Elucidation of the biosynthetic pathways for these compounds would lay a solid foundation for their applications. Zhao *et al*. have identified two distinct biosynthetic pathways for flavones in *S. baicalensis*: the classic flavone biosynthetic pathway active in aerial organs, and the root-specific flavone (RSF) pathway exclusively found in the roots [15]. The classic flavone biosynthesis pathway originates from phenylalanine produced by the shikimate pathway [16]. Phenylalanine undergoes sequential catalysis by a series of enzymes, including phenylalanine ammonia lyase (*Sb*PAL), cinnamate 4-hydroxylase (*Sb*C4H), 4-coumaroyl:CoA-ligase (*Sb*CLL-1), chalcone synthase (*Sb*CHS-1), and chalcone isomerase (*Sb*CHI), ultimately forming naringenin, which serves as a common intermediate for the synthesis of most flavones. The conversion of naringenin to apigenin is catalyzed by flavone synthase II-1 (*Sb*FNSII-1), and subsequently apigenin is hydroxylated by flavone 6-hydroxylase (*Sb*F6H) to produce scutellarein [15, 17]. The biosynthetic pathway of specialized RSFs that have evolved also utilize phenylalanine to produce pinocembrin. Firstly, phenylalanine as a substrate is catalyzed into cinnamic acid by *Sb*PAL. Then, a specific cinnamate CoA ligase (*Sb*CLL-7) is recruited to form cinnamoyl-CoA, followed by condensation with malonyl-CoA by a chalcone synthase (*Sb*CHS-2) and *Sb*CHI to form pinocembrin. Pinocembrin serves as a substrate for flavone synthase isoform FNSII-2 (*Sb*FNSII-2), which specifically converts pinocembrin to chrysin. Chrysin is then hydroxylated by flavone 6-hydroxylase (*Sb*F6H) and flavone 8-hydroxylase (*Sb*F8H) in bifurcating pathways, resulting in the production of baicalein and norwogonin, respectively [15, 17]. Norwogonin is further modified by an 8-*O*-methyltransferase (*Sb*PFOMT5) to form wogonin [18, 19]. Additionally, flavonoid 7-*O*-glucosyltransferase (*Sb*UBGAT) catalyzes the glycosylation of baicalein using UDP-glucuronic acid as the sugar donor, producing baicalin [20, 21]. Furthermore, recent research has identified certain members of the MYB and WRKY gene families as key transcription factors (TFs) that play a crucial role in promoting flavone biosynthesis in *S. baicalensis*[22–24].

The functional identification of biosynthetic genes for bioactive compounds has provided a crucial foundation for further investigations into the biosynthesis and regulation of these natural products in *S. baicalensis * [15, 17]. Through the application of synthetic biology, baicalein and scutellarein production has been successfully achieved *in vivo* using *Escherichia coli* and *Saccharomyces cerevisiae* as host organisms [25–28]. However, the discovery and optimization of functional enzymes involved in biological component synthesis constitute an important requirement for the metabolic engineering of these compounds. It is important to address the above issues for identifying genes or enzymes with functional characterization based on high­throughput omics data screening. The genome and transcriptome of *S. baicalensis* have been sequenced and extensively characterized over the past 10 years [14, 18, 29–31]. Limited studies of the proteome as the main executer of most biological processes in *S. baicalensis* have yielded promising insights.

Protein phosphorylation is a frequently occurring and abundant type of post-translational modification in eukaryotic systems [32, 33]. It serves as a pivotal molecular process that mediates protein function and turnover in response to diverse intracellular and extracellular stimuli. The phosphorylation status modulates protein activity, stability, and structure, while also controlling subcellular distribution and regulating interactions with other proteins. Consequently, phosphorylation-based protein regulation is vital for essential processes like metabolism and development [32]. Protein phosphorylation and dephosphorylation are dynamic processes catalyzed by clusters of enzymes known as kinases and phosphatases, respectively. These enzymes modify the activity of proteins by adding or removing phosphate groups from specific amino acid residues. In *Arabidopsis*, nearly 4% of protein-encoding genes encode kinases [34], highlighting the significance of phosphorylation-based protein regulation [35]. Despite the recent increase in large-scale phosphoproteomic studies in plant species [35–37], only sporadic studies have indicated that TFs or key enzymes are phosphorylated and further activate the downstream genes regulating the accumulation patterns of flavonoids [35, 38, 39], and the identification of phosphoproteins and phosphosites in medicinal plants is far from comprehensive.

In this study we utilized mass spectrometry and RNA sequencing (RNA-seq) analyses to generate the first integrated transcriptomic, proteomic, and phosphoproteomic atlas of *S. baicalensis*. Additionally, we conducted a detailed dissection of plant organs to demonstrate how this comprehensive molecular resource can facilitate the investigation of the function of individual protein families or entire pathways across multiple omics levels. These findings offer valuable insights for molecular-assisted breeding of important TCM resources, genome editing, and improved understanding of the molecular mechanisms underlying the chemical diversity of active compounds.

## Results

### Multi-omics atlas of *S. baicalensis*

**Figure 1:**
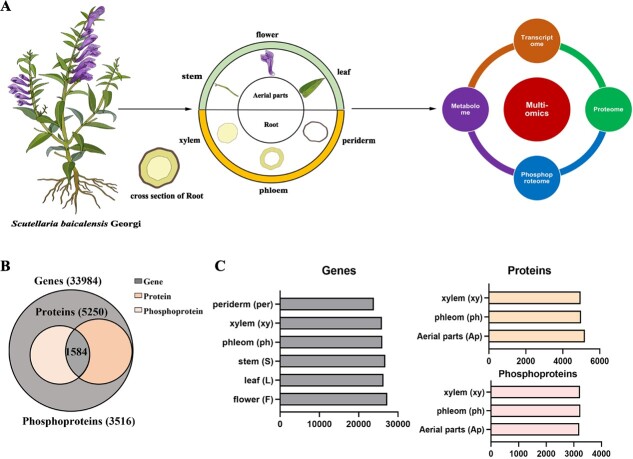
Schematic illustrating the study design and multi-omics dataset of *S. baicalensis*. **A** Schematic of tissue samples analyzed from *S. baicalensis* and multi-omics design. **B** Total number and overlap of identified genes, proteins, and phosphoproteins in the transcriptome, proteome, and phosphoproteome datasets. **C** Number of identifications at the protein, phosphoprotein, and transcript levels for all tissues (*n* = 3 measurements per tissue). Aerial parts include flower, leaf, and stem.

The multi-omics atlas aims to provide a comprehensive and detailed understanding of the interactions and relationships between different biological molecules in a specific system or organism, especially for non-model plants. Considering this, we designed sample categories and an experimental workflow employing a reproducible analytical approach (Fig. 1A). The entire plant was meticulously divided into flowers, leaves, stems, xylem, phloem, and periderm for comprehensive analyses encompassing transcriptomics, proteomics, phosphoproteomics, and metabolomics. Given the constraints posed by limited peptide information and spectral libraries specific to *S. baicalensis*, the atlas was constructed utilizing the full-length genome dataset. Utilizing this database, a comprehensive set of data was generated, encompassing a total of 33 984 genes, 5250 proteins, and 3516 phosphoproteins harboring 7299 quantified phosphorylation sites (P-sites) across multiple samples (Fig. 1B). All the genes, proteins, and phosphorylation sites were categorized for each tissue, revealing that they share similar quantities (Fig. 1C). The atlas study can contribute to the advancement of high-throughput quantitative mass spectrometry-based protein assays, a particularly valuable resource for *S. baicalensis* research given the limited availability of antibodies.

### Organ-specific localization of active components involved in flavonoid biosynthesis

**Figure 2:**
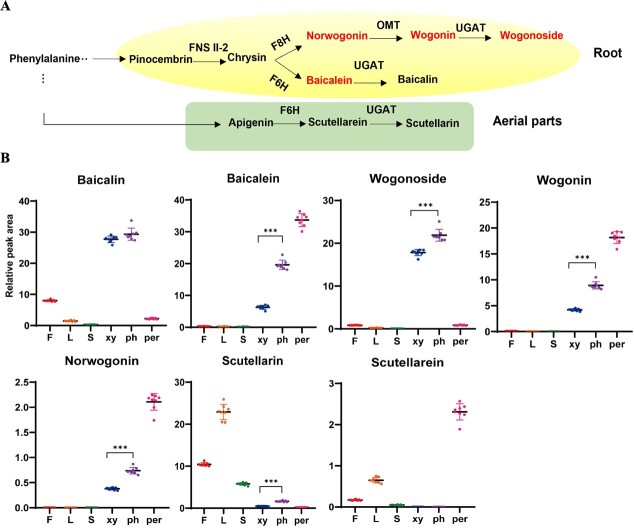
Accumulation profiling of active components participating in flavonoid biosynthesis. **A** Distinct biosynthetic pathway of main flavonoids between aerial parts and root in *S. baicalensis*. FNSII-2, flavone synthase II; F6H, flavone 6-hydroxylase; F8H, flavone 8-hydroxylase; UGAT, glucuronosyltransferases; OMT, flavone 8-*O*-methyltransferase. **B** Contents of flavonoids and their glycosides detected in the tissue metabolome (*n* = 8). F, flower; L, leaf; S, stem; ph, phloem; xy, xylem; per, periderm. ^*^P ≤ 0.05, ^***^P ≤ 0.001.

The main bioactive compounds of *S. baicalensis* include baicalein, scutellarein, norwogonin, wogonin, and their glycosides (baicalin, scutellarin, norwogonoside, and wogonoside). They are distributed to two distinct flavonoid biosynthetic pathways, as shown in Fig. 2A; the lower pathway on a green background represents the classic flavone biosynthetic pathway active in aerial organs, while the upper pathway represents the evolved pathway specific to the root. We collected samples from the flower, leaf, stem, phloem, xylem, and periderm tissues of *S. baicalensis* to determine the accumulation of these active compounds. As shown in Fig. 2B, baicalein, norwogonin, wogonin, baicalin, and wogonoside accumulated mainly in the root of *S. baicalensis*, while scutellarin and scutellarein were distributed in the aerial parts (stem, leaf, and flower). The result is consistent with a previous study [14]. Interestingly, among the root-specific flavones, baicalein and wogonin were found to have the highest accumulation in the periderm, while their glycosides, baicalin and wogonoside, exhibited the highest levels in the phloem tissues. This observation suggests that the UDP-glycosyltransferases (UGATs) responsible for these processes may have higher enzymatic activity in the phloem than in the periderm.

### mRNA quantitative expression landscapes

To elucidate the root cause of the formation of two distinct pathways in *S. baicalensis*, we performed RNA-seq to obtain a coding sequence database and constructed a high-throughput transcriptomic map covering flowers, leaves, stems, phloem, xylem, and periderm. More than 40 000 000 clean reads were obtained in the six sample tissues with three biological replicates (Supplementary Data Table S1). The assembled genes were translated into coding sequences, which served as a proteomics database that can be used to construct a library for non-model plants.

**Figure 3:**
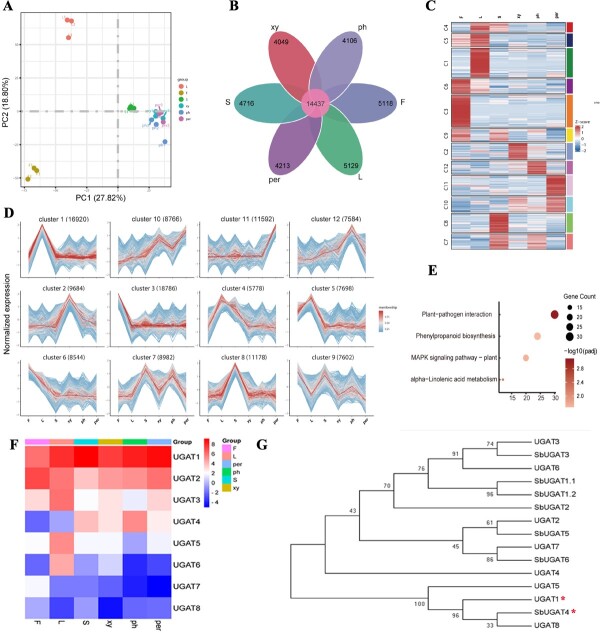
Transcriptomic profiling of tissue from flower, leaf, stem, phloem, xylem, and periderm in *S. baicalensis*. **A** PCA result of all genes obtained from RNA sequencing of samples tissues, where each dot represents the combined expression data for all genes from a single tissue sample. **B** Venn diagram of the number of genes that are uniquely expressed within each group and co-expressed in multiple groups. **C** Differential expression gene clustering heat map produced by the Mfuzz R package. All genes with significantly altered expression (threshold of |log2(FoldChange)| ≥ 1, *P*_adj_ ≤ 0.05) are displayed and clustered using the log_2_(FPKM + 1) value. Color ranging from red to blue indicates log_2_(FPKM + 1) values ranging from large to small. **D** Overall cluster results of FPKM analysis using the log_2_(FPKM + 1) value. **E** KEGG pathways of genes from cluster 10 matrix (*P*_adj_ ≤ 0.05) based on gene expression differences across six tissues. **F** Expression pattern of UGAT from the transcriptomic database of *S. baicalensis*. **G** Phylogenetic tree analysis of UGAT protein sequences. Neighbor-joining was employed to construct this tree with 1000-replicate bootstrap. The gene loci of *Sb*UGAT1.1, *Sb*UGAT1.2, *Sb*UGAT2, *Sb*UGAT3, *Sb*UGAT4, *Sb*UGAT5 and *Sb*UGAT6 are *Sb*01g50771.p1, *Sb*01g50771.p2, *Sb*01g50791, *Sb*01g51711, *Sb*01g56811, *Sb*01g56821 and *Sb*09g13460.

The genome of *S. baicalensis* was downloaded from the China National Center For Bioinformation (https://www.cncb.ac.cn/?lang=en) with accession number GWHBJEC00000000 under BioProject PRJCA009554 [31]. Depending on the genome of *S. baicalensis*, ~90% of genes were mapped, whereas the mapping ratio was <75% in periderm tissue (Supplementary Data Table S2). To assess sample integrity across all six tissue types, we performed principal component analysis (PCA) and Pearson correlation coefficient analysis and found that samples from flower, leaf, and stem groups showed the great separation (Fig. 3A, Supplementary Data Fig. S1A), thus verifying sample integrity. No clear separation was observed among the samples from root tissues, including the phloem, xylem, and periderm, as shown in Fig. 3A. The co-expression analysis identified 14 437 genes that were commonly expressed across all samples (Fig. 3B). To compare the abundance of differential expression genes (DEGs) in different tissues, differential analysis was performed and numbers of DEGs were also listed in detail (Supplementary Data Fig. S1B). In the comparison of the aerial parts, there were ~8000–10 000 different genes, while in the root there were fewer, only ~3000. When comparing xylem and phloem, there were only 624 genes that differed (Supplementary Data Table S3). For more information, cluster analysis was carried out on different gene sets and genes with similar expression patterns were gathered together by hierarchical clustering using the Mfuzz package (Fig. 3C). A total of 12 clusters were drawn, with genes highly expressed in the root part mainly enriched in cluster 10 (Fig. 3D). KEGG (Kyoto Encyclopedia of Genes and Genomes) enrichment analysis showed that plant–pathogen interaction, phenylpropanoid biosynthesis, and the MAPK signaling pathway (Fig. 3E) ranked as the top three (*P*_adj_ ≤ 0.05) in cluster 10, whereas the enrichment pathway in cluster 5, which highly accumulated scutellarin, was mainly involved in circadian rhythm (Supplementary Data Fig. S1C). The results indicated that the evolved RSF pathway depends on plant–pathogen interaction, phenylpropanoid biosynthesis, and the MAPK signaling pathway at transcriptional level, whereas the classic flavone biosynthetic pathway formed in aerial organs is associated with the circadian rhythm. In addition, eight UGAT genes were identified from the transcriptomic database (Fig. 3F). UGAT1, which was highly expressed in all sample tissues, was 98% identical to *Sb*UGAT4 identified by Zhao’s team [20]. As shown in Fig. 3G, a phylogenetic tree analysis showed that UGAT1 had a close evolutionary relationship with *Sb*UGAT4, effectively catalyzing flavones into their glycosides in the root. Based on the phylogenetic tree analysis and multiple sequence alignment, UGAT1 shared the same amino acid sequence as *Sb*UGAT4, excluding the first 53 amino acids (Supplementary Data Fig. S2). Additionally, it has been demonstrated that *Sb*UGAT4 can use UDP-glucuronic acid as the sugar donor and catalyze baicalein to baicalin with *K*_m_ 10.20 μM [20]. Based on the experimental data of the metabolite content measurement in Fig. 2B, compared with xylem, the main flavone glycosides, baicalin and wogonoside, were highly accumulated in phloem. Therefore, we believe that UGAT1, like SbUGAT4, plays a potential role in the accumulation of flavone glycosides in the phloem.

### Global analysis of proteomic landscape

Using the genome of *S. baicalensis* [31], an annotated coding sequence library was generated and a substantial number of 30 100 amino acid sequence entries were corroborated. As expected, a total of 5250 proteins and 28 692 peptides were identified depending on the data-independent acquisition (DIA) method, whereas 5064 proteins were comparable for quantitative analysis, as shown in Supplementary Data Fig. S3A. From Pearson’s correlation coefficient, we found that sample groups displayed obvious clustering (Supplementary Data Fig. S3B). Based on common annotated databases, including COG/KOG, DOMAIN, KEGG, and GO (Gene Ontology), 3770, 2961, 2199, and 2006 proteins were mapped, respectively (Supplementary Data Fig. S3A, Supplementary Data Table S4).

**Figure 4:**
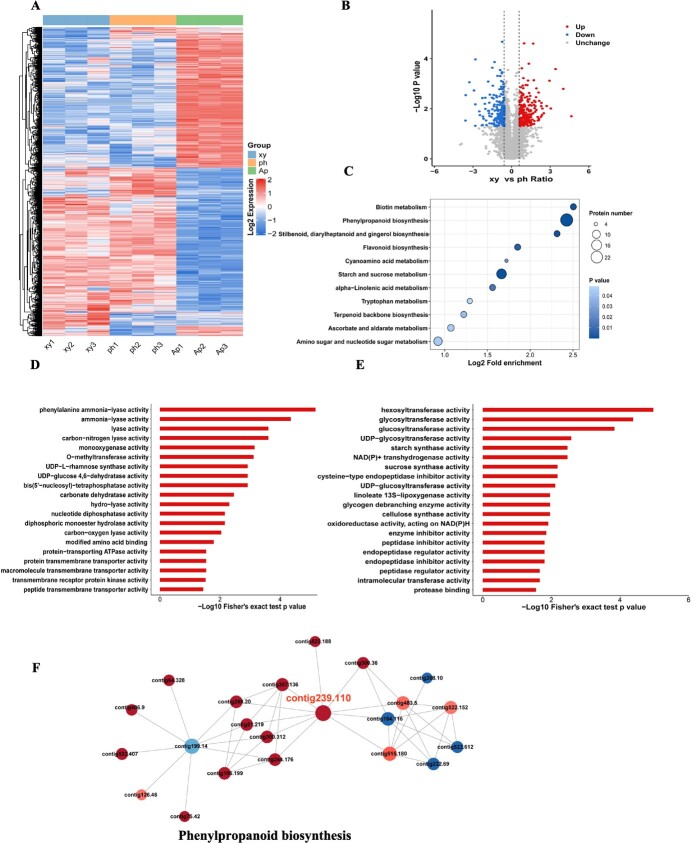
Overview of proteomics analysis between xylem and phloem of *S. baicalensis*. **A** Expression heat map of differential proteins across multiple tissues, |log_2_(FoldChange)| ≥ 0.6 and *P* ≤ 0.05. Red color indicates upregulation and blue color indicates downregulation. **B** Volcano plot of different proteins between xylem and phloem. Red dots represent upregulated proteins and blue dots represent downregulated proteins, while the gray part represents unchanged proteins. **C** KEGG enrichment of different proteins between xylem and phloem (*P* ≤ 0.05). **D** Molecular function classification of upregulated proteins in xylem. **E** Molecular function classification of downregulated proteins in phloem. **F** Network analysis of differential proteins in xylem versus phloem (confidence score >0.7). Each circle represents a distinct pathway annotation. Red and blue dots indicate enrichment of upregulated and downregulated proteins, respectively, in that pathway annotation. The size of the dot indicates the number of proteins interacting with it. The thickness of the color represents the multiple of difference. Contig 239.110, 4-coumarate:CoA ligase 1.

Subsequently, we identified differential expression proteins in each tissue with a cutoff of |log_2_(FoldChange)| ≥ 0.6 and *P* value ≤0.05. We found that 1075 proteins were upregulated and 1074 proteins were downregulated in phloem versus aerial parts (Supplementary Data Fig. S3C), whereas 1072 proteins were upregulated and 993 proteins were downregulated in xylem versus aerial parts (Supplementary Data Fig. S3D). The heat map in Fig. 4A shows that the proteins in the aerial parts vary greatly compared with those in the phloem and xylem. While the phloem and xylem share similar protein trends, they also have unique differential proteins. It was a remarkable fact that 205 upregulated and 180 downregulated proteins existed in xylem versus phloem tissues, and details of the differential proteins can be seen in Fig. 4B and Supplementary Data Table S5.

The KEGG analysis indicated that all of the differential proteins were enriched in 59 pathways. Among these pathways, phenylpropanoid biosynthesis was the focus, with 25 differential proteins ranked first, whereas flavonoid biosynthesis had 6 differential proteins, as shown in Fig. 4C and Supplementary Data Table S6. The majority of upregulated proteins have molecular functions related to phenylalanine ammonia-lyase (PAL) activity and ammonia-lyase activity, while the majority of downregulated proteins are associated with hexosyltransferase activity and glycosyltransferase (GT) activity (Fig. 4D and E). Protein domain results indicated that peroxidase, cytochrome P450 and *O*-methyltransferase were significantly changed in xylem versus phloem (Supplementary Data Fig. S3E). The protein–protein interaction network analysis revealed that phenylpropanoid biosynthesis was the most significant component involved in molecular functions and physiological regulation in the root. Most of the proteins involved in the phenylpropanoid biosynthesis pathway were upregulated. Of particular note is that contig 239.110 is a 4-CoA ligase enzyme that predominantly interacts with PAL enzymes (Fig. 4F). Together, these results indicate that redox reaction of phenylpropanoid and flavonoid biosynthesis involving peroxidase, cytochrome P450, *O*-methyltransferase, and the electron transport chain promoted secondary metabolite biosynthesis in roots, leading to the compartmentalization of secondary structure and further physiological improvement.

### Transcriptome and proteome global association analysis in phloem versus xylem

**Figure 5:**
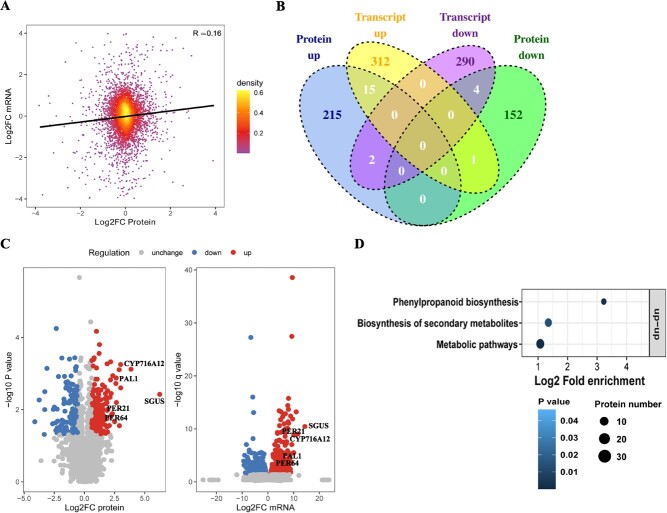
mRNA–protein correlations highlight the regulation pathway of active compound accumulation in phloem. **A** Scatter plot of transcripts and their corresponding protein expression. **B** Venn plots of differential transcripts and proteins. Benjamini–Hochberg calibration was done for *P* value. Significantly differentially expressed transcripts were defined as |log_2_(FoldChange)| ≥ 1 and *P*_adj_ < 0.05. Significantly differentially expressed proteins were set using a cutoff of |log_2_(FoldChange)| ≥ 0.6 and *P* ≤ 0.05. **C** Volcano map showing detailed distribution of differential expression proteins and transcripts. PAL, phenylalanine ammonia-lyase; SGUS, baicalin-β-d-glucuronidase; PER, peroxidase; CYP, cytochrome P450 proteins. **D** Two-tailed Fisher’s exact test to test for enrichment of differentially expressed transcripts and proteins. Pathways with a corrected *P*-value ≤0.05 were considered significant.

To delineate the distinction between phloem and xylem, we conducted a comparative analysis of transcriptome and proteome quantification. A total of 4455 genes were quantified at both the transcriptome and proteome levels. A scatterplot depicting the relationship between transcripts and the expression of their corresponding proteins revealed a positive correlation, indicating that gene expression is associated with protein expression (*R* = 0.16) (Fig. 5A). These results indicated that RNA abundance does not reliably predict protein abundance, suggesting that post-transcriptional regulation or post-translational modification may exert a potential influence on the functional aspects of the metabolite-protein network. 328 upregulated transcripts and 232 upregulated proteins were identified , 15 of which are intersections as co-upregulated genes. These 15 co-upregulated genes include PAL1 (O23865, *Daucus carota*), SGUS (Q9LRC8, *S. baicalensis*), PER21 (Q42580, *Arabidopsis thaliana*), PER64 (Q43872, *A. thaliana*), and CYP716A12 (Q2MJ20, *Medicago truncatula*), which are extensively involved in biosynthetic pathways of secondary metabolites (Fig. 5B and C). KEGG cluster analysis also displayed 15 co-upregulated genes and proteins that mostly execute their function in phenylpropanoid biosynthesis (Fig. 5D). Above all, the association analysis revealed that high accumulation of bioactive metabolites in phloem was likely to be regulated by phenylpropanoid biosynthesis, especially for the PAL, peroxidase (PER), and CYP450 gene families. It is noteworthy that recent studies indicate an absence of correlation between transcript and protein abundance, suggesting that changes in transcription may not reliably predict protein-level regulation. It is currently unclear whether this lack of coincident regulation also pertains to flavonoid-related proteins controlled by post-translational modification.

**Figure 6:**
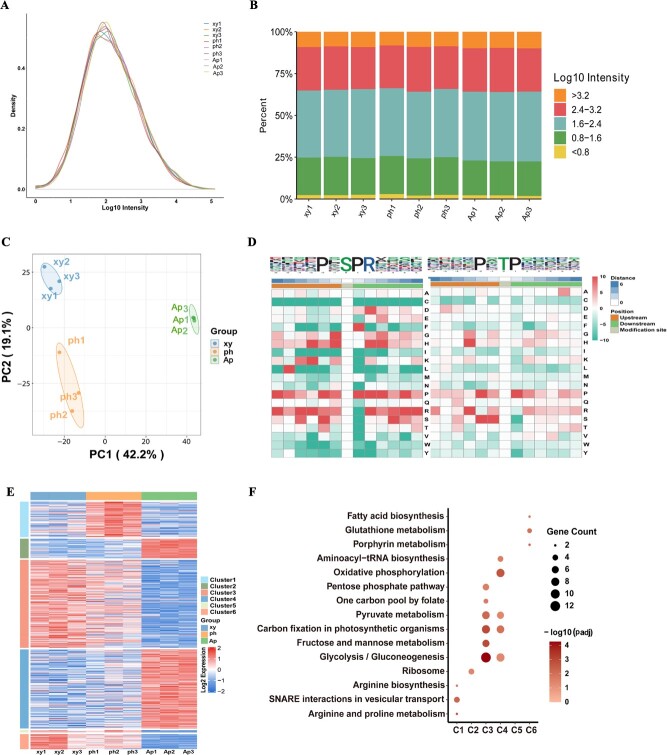
Phosphoproteome overview of *S. baicalensis.***A**, **B** Distribution of phosphopeptide abundance in the DIA data. Data are average expression of the nine tissues profiled. **C** PCA analysis of all modified sites across multiple tissue samples in *S. baicalensis*. **D** Motif characterization of phosphopeptides by MoMo with the cutoff of peptide counts of motif >20 and *P* < 0.000001. The phosphorylation modification residue refers to six sites upstream and downstream, respectively. **E** Expression mapping of phosphopeptides across phloem, xylem, and aerial part tissue, |log_2_(FoldChange)| ≥ 0.6 and *P* value ≤ 0.05 were set as the significance threshold. **F** Cluster enrichment results aligned with expression patterns of differential proteins, as illustrated in Fig. 5E.

### Phosphoproteomic landscape of *S. baicalensis*

Understanding kinase–substrate relationships is crucial for comprehending signaling cascades or pathway activities. Scientists often employ a computational approach to obtain this information, as it can be difficult to discover experimentally [35]. As is well known, the key adjacent residues, called ‘motifs’, that cause specific enzyme substrate interactions are the focus of research, because the partial biochemical preference of an enzyme for a given substrate may be determined by the residues around the modification sites.

Employing a well-established protocol for deep-scale phosphoproteomic analysis, we conducted global phosphopeptide measurements across the nine samples. Our analysis identified 7299 phosphosites on 3516 unique proteins within the dataset. Notably, the distribution of intensity values for modification sites remained consistent across various samples (Fig. 6A and B). Furthermore, PCA clustering based on phosphoproteomic profiles demonstrated distinct expression patterns of modified sites among different tissues (Fig. 6C). Leveraging the available phosphoproteomic data, we utilized the motif-X algorithm to discern 42 phosphorylation motifs, categorized into ‘serine-directed’, ‘threonine’, or ‘tyrosine’ motif classes (Supplementary Data Table S7). Notably, two distinctive sequence types, namely xxxxPx_S_PRxxxx and xxxxPx_T_Pxxxxx, emerged as unique motifs in *S. baicalensis* (Fig. 6D). A total of 3516 phosphoproteins were identified and mapped to 1472 proteins through phosphoproteomic analysis. These proteins were subsequently annotated in the COG/KOG, Domain, KEGG, and GO databases, respectively (Fig. 1B, Supplementary Data Fig. S4A). Details of 235 phosphopeptides are listed in Supplementary Data Table S8 and a radar map of the top 30 is shown in Supplementary Data Fig. S4B. Expression abundance analysis revealed 235 phosphopeptides that mapped to 207 proteins and exhibited significant differential changes, which were categorized into six clusters (Fig. 6E). Proteins exhibiting high expression in the aerial parts predominantly clustered in oxidative phosphorylation and glycolysis/gluconeogenesis. Those highly expressed in the phloem were enriched in synaptosome-related interactions associated with vesicular transport, whereas proteins with elevated expression in the xylem primarily clustered in glycolysis/gluconeogenesis and carbon fixation in photosynthetic organisms (Fig. 6F).

The statistical analysis of differentially expressed peptides and corresponding proteins between groups revealed that 101 phosphopeptide sites were upregulated and 134 were downregulated in xylem versus phloem. Subsequently, this list of 207 proteins underwent KEGG pathway classification analysis, GO secondary classification, subcellular localization classification, and protein domain enrichment analysis. According to KEGG analysis, glycolysis/gluconeogenesis, pyruvate metabolism, and carbon fixation in photosynthetic organisms were identified as the major clustering for differential proteins in xylem, phloem, and aerial parts. For a more comprehensive understanding of the biological processes of these phosphoproteins, we conducted molecular function and domain analyses revealing the significant involvement of ATPases and hydrolases in glycolysis/gluconeogenesis (*P* ≤ 0.05, Fisher’s exact test) (Supplementary Data Fig. S4C). Moreover, 32.37 and 24.64% of the differential proteins were localized in the nucleus and chloroplast, respectively, underscoring their significant biological functions. These findings highlight the robust conservation of post-translational protein modifications as a mechanism to generate functional diversity from a limited number of genes and proteins.

### Post-translational protein modification regulation in flavonoid biosynthesis

**Figure 7:**
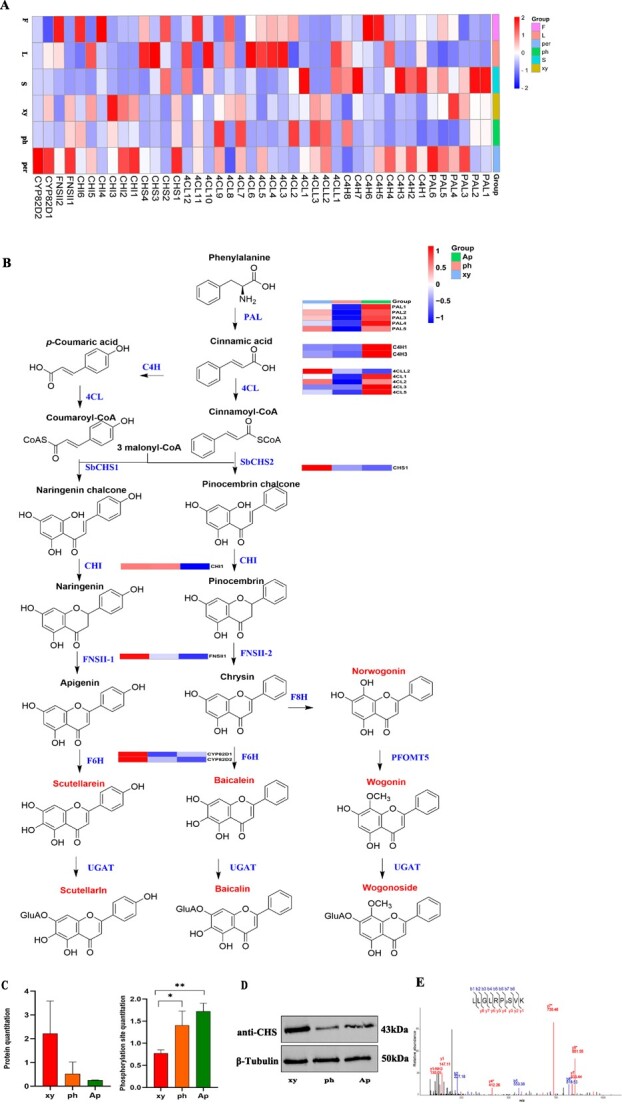
Gene and protein expression regulation involved in flavonoid biosynthesis pathway. **A** Expression map of all genes identified participating in the flavonoid biosynthesis pathway. PAL, phenylalanine ammonia-lyase; C4H, cinnamate 4-hydroxylase; 4CL, 4-CoA ligase; CHS, chalcone synthase; CHI, chalcone isomerase; FNSII, flavone synthase II; F6H, flavone 6-hydroxylase; F8H, flavone 8-hydroxylase. **B** Proposed pathways for synthesis of flavonoids in *S. baicalensis*. Proteins reported in the proteomics dataset in this study are labeled in the expression map. UGT, glycosyltransferase; UGAT, glucuronosyltransferases; OMT, flavone 8-*O*-methyltransferase. **C** Protein quantitation and phosphorylated site quantitation of CHS1. ^*^P ≤ 0.05, ^**^P ≤ 0.01. **D** Protein expression result of CHS1 in xylem (xy), phloem (ph) and aerial parts (Ap) by western blotting with β-tubulin as internal control. **E** Phosphorylation of CHS1 *in vivo* confirmed by PRM analysis. ^*^Neutral loss, 98 *m*/*z* (H3P4).

As previously mentioned, the biosynthesis pathway of flavonoids in *S. baicalensis* is currently believed to be divided into two pathways: the classic biosynthesis pathway in the aerial parts and the evolved biosynthesis pathway in the root. To delineate these pathways, we extracted and identified the structural enzymes from transcriptome databases. Subsequently, we classified these enzymes into seven categories, as illustrated in Fig. 7A. Detailed information for the 43 genes is listed in Supplementary Data Table S9, including the PAL, C4H, 4CL, CHS, CHI, FNSII (CYP93B), and F6H/F8H (CYP82D) gene families. Utilizing mass spectrometry proteomics technology, we identified a total of 17 proteins corresponding to the 43 genes. The upstream protein PAL is implicated within both pathways, exhibiting high expression primarily in the aerial parts. Furthermore, upstream enzymes C4H and 4CL in the flavonoid pathway are also predominantly expressed in the aerial parts. After crossing the bridge protein of CHS in the flavonoid biosynthesis pathway, the expression of downstream proteins is predominantly concentrated in the xylem, as shown in Fig. 7B.

Chalcone synthase serves as the rate-limiting enzyme in the flavonoid biosynthesis pathway, and its characteristics and activity directly influence the flux and accumulation of downstream secondary metabolites. By sequence alignment, we found that CHS1 in this study and *Sb*CHS2 (protein accession number, AMW91736), reported previously, have 99% sequence similarity, with only three amino acids differing (Supplementary Data Fig. S5). *Sb*CHS2 has been reported to exhibit high expression in the root and to display catalytic activity, particularly in the biosynthesis of pinocembrin chalcone, thereby playing a key role in RSF biosynthesis [15]. According to the proteomics data, CHS1 expression was primarily in the xylem initially, whereas expression in the phloem and aerial parts was comparatively lower (Fig. 7C). The western blot shown in Fig. 7D confirmed this finding. Depending on the 4D-DIA phosphoproteomics data, CHS1 was found to be phosphorylated at a serine site (153) and further confirmed by parallel reaction monitoring (PRM) liquid chromatography–mass spectrometry (LC–MS) as shown in Fig. 7E. Moreover, following the phosphorylation modification, CHS1 exhibited enhanced expression, spreading from the xylem to the phloem and aerial parts, thus causing downstream metabolite flow to the phloem, as depicted in Fig. 7C. This differs from the protein expression pattern. This study suggests that CHS1 is phosphorylated and further activates the key downstream gene regulating the accumulation patterns of flavonoids.

### Protein phosphorylation alters the flavone pathway

**Figure 8:**
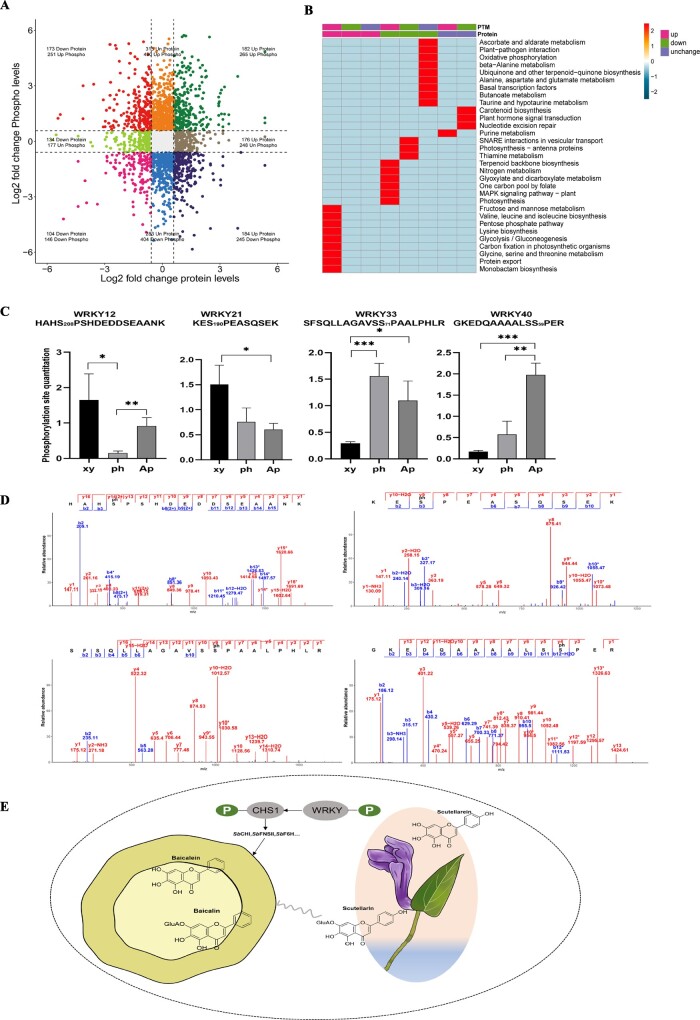
Association analysis of the proteome and phosphoproteome reveals the production of distinct flavone pathways. **A** Quadrant chart of differential proteins and phosphoproteins in phloem versus aerial parts. |log_2_(FoldChange)| ≥ 0.6 was set as a threshold. **B** KEGG enrichment analysis comparing phloem with aerial parts. **C** Phosphorylated WRKY transcription factor expression profiling ^*^P ≤ 0.05, ^**^P ≤ 0.01, ^***^P ≤ 0.001. **D** MS^2^ pattern of four WRKY TF’s phosphorylation sites by PRM. **E** Hypothetical model for TF *Sb*WRKY and CHS’s phosphorylation to distinct flavone pathways between the aerial part and root in *S. baicalensis*.

Association analysis of the proteome and phosphoproteome allows the key protein loci associated with the studied traits to be found, reducing the number of candidate protein loci. As the phloem was the significant second structure of root with bioactive component accumulation, we compared proteome and phosphoproteome profiling in phloem and aerial parts to explore the effect of phosphorylation on distinct flavone pathways. A total of 1584 proteins were identified at protein and phosphoprotein levels. Among these, 542 proteins were upregulated in phloem, while 510 phosphoproteins exhibited change. In contrast, 411 proteins were downregulated, with alterations observed in 397 phosphoproteins, as depicted in the quadrant chart in Fig. 8A, with a cutoff of |log_2_(FoldChange)| ≥ 0.6. KEGG enrichment analysis revealed that protein export and valine, leucine, and isoleucine biosynthesis were the top two significantly ranked phosphorylated pathways positively regulating protein expression in the phloem, as part of flavonoid biosynthesis. Additionally, glycolysis/gluconeogenesis and carbon fixation in photosynthetic organisms exhibited the highest number of proteins in this context. Conversely, in aerial parts, the phosphorylated pathways primarily involved in nitrogen metabolism, the MAPK signaling pathway, and glyoxylate and dicarboxylate metabolism were identified as negatively regulating the expression of responsive proteins (Fig. 8B).

In eukaryotes, TFs play a vital role in plant growth, development, and stress responses via the self-regulation and expression regulation of target genes [24]. The discovery of TFs involved in flavonoid regulation is beneficial to the study ofheterologous synthesis of flavonoids.. Myeloblastosis (MYB) proteins can be divided into R1-, R2R3-, R1R2R3- (3R-), and 4R-MYB proteins [40, 41], among which R2R3-MYB TF subgroups are involved in mostly secondary metabolite biosynthesis. In a previous study, *Sb*MYB3, functioning as a positive regulator, was shown to bind to the promoter of *Sb*FNSII-2, thereby promoting RSF biosynthesis in *S. baicalensis* [22]. *Sb*MYB12, a nuclear-localized transcription activator that binds to the *Sb*CCL7-4, *Sb*CHI-2, and *Sb*F6H-1 promoters, positively regulates the generation of baicalin and wogonoside [23].

Depending on the transcriptome of different tissues of *S. baicalensis*, we identified TFs that potentially regulate the synthesis of RSFs. In total, 117 R2R3-MYB TFs were found (Supplementary Data Fig. S6A). We constructed a phylogenetic tree using protein sequences of MYBs. Phylogenetic analysis indicated that MYB67, MYB96, MYB113, MYB102, and MYB90 belong to subgroups 14, 4, 20, 20, and 22 [17], which are close to *Sb*MYB1, *Sb*MYB2, *Sb*MYB3, *Sb*MYB4, and *Sb*MYB6, respectively. MYB48 has a close phylogenetic relationship to *Sb*MYB5, which is similar to *At*MYB82 [42] (Supplementary Data Fig. S7). However, no phosphorylation site of MYB was identified. We analyzed the WKRY TF family, which is involved in the regulation of a variety of stress and immune response in many plant species [43, 44]. Seventy-one WRKY genes were identified and most of them are highly expressed in the aerial parts; this is consistent with a previous study (Supplementary Data Fig. S6B) [42]. Four WRKY TFs were found to have four phosphorylation sites; phosphorylation of WRKY40 was expressed in aerial parts when WRKY12 and WRKY21 were phosphorylated in xylem and WRKY33 was highly expressed in aerial part and xylem(Fig. 8C). By PRM experiments, the phosphorylation sites of four WRKYs were reconfirmed by mass shift calculation between b and y ions (Fig. 8D).

As shown in Fig. 2B, it is worth noting that scutellarin and baicalin accumulated in both the aerial parts and the roots, which means two distinct pathways did not exist separately, suggesting a possible interaction between the classic and evolved pathways that requires further investigation (Fig. 8E). Meanwhile, phosphorylation modification of functional proteins and TFs may play a significant role in the biosynthetic diversity of natural products.

## Discussion

*Scutellaria baicalensis*, a widely used medicinal plant, has had its active ingredient biosynthesis pathway largely resolved in recent years with the development of genome and transcriptome sequencing technologies [45, 46]. The heterologous synthesis of some important components has been achieved through synthetic biology methods, providing more alternative pathways for acquiring active ingredients [25, 27, 28]. Reclaiming the unexplored land of *S. baicalensis* has become a new scientific research proposition. In this study, we proposed a macroscopic mapping strategy based on cutting-edge proteomics methods, and verified and expanded the information on the expression regulation of a series of genes. For instance, CHS1, highly homologous to the reported *Sb*CHS2 [15], was highly expressed in the xylem and involved in the synthesis of the active ingredient in the root. However, its phosphorylation modification reduced the expression of the modified peptide in the xylem. Conversely, phosphorylation modification decreased the expression of the modified peptide in the xylem and increased its expression in both the phloem and aboveground parts. UGAT1 is highly homologous to the reported *Sb*UGAT4 [20], which was generally highly expressed across multiple tissues at the transcriptome level, but the protein is highly expressed only in roots while phosphorylation modification decreased its expression in the xylem. These results suggested that post-translational modification plays an important role in the execution of protein function.

Phosphorylation modifications were widely present in all tissues of *S. baicalensis*, being enriched in in glycolytic and oxidative phosphorylation pathways, with modified proteins accounting for 67% of the total number of proteins identified. In this study we focused more on the phosphorylation modifications of functional enzymes and TFs related to the flavonoid biosynthesis pathway. Among these modified proteins identified based on protein profiling, some of the modified peptides have missing K or R sites, which need to be confirmed by further technical means, such as CHS1, identified in Fig. 7E, which is phosphorylated by S at position 153 but has a missing R site at position 151; this needs further experimental verification. In addition, UGAT1 is a well-established GT that has been reported to play an important function in the biosynthesis of baicalin. For the first time, we report the phosphorylation of UGAT1 at site 33, which obviously affected protein expression. Additionally, we also found many phosphorylation sites of other protein enzymes in the proteomic dataset, but more evidence needs to be provided to demonstrate their function.

As is known well, GTs control the formation of glycosidic bonds in plant secondary metabolism [47]. GTs with catalytic promiscuity are generally recognized as powerful tools in the glycodiversification of natural products, derivatives of which constitute a large proportion of clinical drugs [48]. Mining functional GTs from *S. baicalensis* is critical to synthesize bioactive flavonoid glycosides. Given the reported GTs of *S. baicalensis*, six glucosyltransferases (*Sb*UGTs) could convert baicalein to oroxin A using UDP-glucose as a sugar donor, while four glucuronosyltransferases (*Sb*UGATs) catalyzed baicalein to baicalin using UDP-glucuronic acid as the sugar donor [48]. In this study, a total of eight UGATs were identified while the proteomics study showed that one out of eight UGAT proteins, UGAT1, was expressed. The multiple alignment analysis revealed that these UGATs shared the PSPG (plant secondary product glycosyltransferase) box, which plays a critical role in the recognition of the UDP-glucuronic acid sugar donor (Supplementary Data Fig. S7). The previous site-directed mutagenesis analysis demonstrated that an Arg residue (R) was employed to recognize the UDP-glucuronic acid sugar donor while the corresponding Trp residue (W) has better selectivity for the UDP-glucose donor [49, 50]. UGAT1 shared a very close phylogenic relationship with *Sb*UGAT4, which had high enzymatic activity in catalyzing flavonoids into flavonoid glycosides. Phosphoproteomics analysis showed that UGAT1 was phosphorylated on ser33, significantly affecting protein expression across multiple tissues. These results indicated that phosphorylated UGAT1 may play a potential role in metabolite accumulation in root, especially in phloem. Additionally, there are ample flavonoid C-glycosides or di-C-glycosides in *S. baicalensis*, and the responsible C-GTs remain to be explored in the future.

In our study, the periderm, which is the outer layer of the root and considered a protective structure, was found to contain bioactive compounds, consistent with previous studies in *Salvia miltiorrhiza* [51]. Metabolite analysis revealed that baicalein, norwogonin, wogonin, and scutellarein were prominently detected in the periderm. Additionally, we re-examined highly expressed genes in cluster 11 (Supplementary Data Fig. S1C), which primarily participate in photosynthesis and carbon metabolism pathways unrelated to secondary metabolite biosynthesis. Due to the complex structure of the periderm and its difficulty in being peeled off without contamination from phloem mixtures, the accumulation of bioactive components in the periderm remains to be further discussed.

## Conclusions

Our study utilized mass spectrometry-based proteomics and phosphoproteomics to build a substantial public library to evaluate the metabolic processes and functions of *S. baicalensis*. Using multi-omics technology, the study demonstrated that the classic and evolved flavonoid pathways did not develop alone and had some potential relationship likely relying on post-transcriptional modification. Meanwhile, this research also revealed that phloem is a highly efficient factory for metabolite production in the root, rich in highly effective functional enzymes originating from the phenylpropanoid biosynthesis pathway. This study provides a potential foundation for future optimization and application of *de novo* biosynthesis and germplasm resources in *S. baicalensis*.

## Materials and methods

### Plant materials

*Scutellaria baicalensis* Georgi seeds were purchased from Longxi County, Gansu province of China and were grown in the botanical garden of the Second Military Medical University. Two-year-old plants were cultivated for the following experiments. Flower, leaf, stem, phloem, xylem, and periderm were collected.

### Metabolite measurement

Tissue samples were collected and immediately dried in a heater oven at 40°C. The dried tissues were ground into powder using a mechanical tissue blender, and 40 mg of each sample was prepared well in advance. The metabolites were isolated overnight using 60% methanol and subsequently extracted by ultrasonication for 40 min. Following centrifugation at 12 000 *g* for 10 min at 4°C, the supernatant was meticulously collected for metabolite detection.

Metabolite data were acquired using the Agilent 1290 Infinity system, which incorporated 6545 UHD and Accurate-Mass Q-TOF technologies. Ultra high-performance liquid chromatography (UPLC) analysis utilized an XBridge™ BEH C18 column (2.1 × 100 mm, 2.5 μm, Waters) for reverse phases. Mobile phase A consisted of H_2_O (0.1% vol/vol formic acid), while mobile phase B comprised acetonitrile (0.1% vol/vol formic acid). The gradient conditions were as follows: from 0 to 2 min, 2–2% B; from 2 to 17 min, 2–98% B; from 17 to 19 min, 98–98% B. The flow rate was consistently held at 0.4 ml/min, with an injection volume of 3 μl. The ESI source was utilized for data acquisition of mass spectrometry, and the source parameters were set as follows: capillary voltage at 4 kV in positive mode and 3.5 kV in negative mode, fragmentary voltage at 120 V, skimmer voltage at 60 V, dryer flow rate at 11 l/min, gas temperature at 350°C, and nebulizer pressure at 45 psi. Mass ranges of *m*/*z* 50–1500 were acquired in both positive and negative ion mode. We used 121.0509 and 922.0098 in positive mode, as well as 112.9855 and 1033.9881 in negative mode, as reference ions. Normalization was facilitated with the internal standard 2-chloro-l-phenylalanine. A 2-μl aliquot from each individual sample was combined to create a quality control sample, enabling the assessment of system stability throughout the experimental process. Standard compounds were injected one by one under the same condition for relative quantification. A MassHunter B.07 version workstation was used to display spectrums and convert raw data into.mzdata format to calibrate and integrate peaks. Metabolite identification was processed with the MassHunter Profinder 10.0 search tool with a home-built database.

### RNA-sequencing

Total RNA was meticulously extracted using TRIzol reagent (Invitrogen) in accordance with the manufacturer’s instructions. Subsequent assessment of RNA integrity employed the RNA Nano 6000 Assay Kit (Agilent Technologies, USA). In a sequential process, mRNA was purified and enriched using poly-T oligo-attached magnetic beads. Following this, first-strand cDNA and second-strand cDNA were synthesized employing a standardized method. The quality of the RNA-sequencing library was assessed with an Agilent Bioanalyzer 2100 system (Agilent Technologies, USA). Sequencing was carried out on a Novaseq 6000 with PE-150 (Illumina, USA), generating paired-end reads of 150 bp.

Paired-end clean reads were aligned to the *S. baicalensis* reference genome (Bioproject PRJCA009554) using HISAT2 (v2.0.5), which incorporated all splice junctions derived from the gene model annotation file. Quantification of mRNA expression was subsequently performed with HTSeq (version 0.9.1) using the aligned reads. The mapped reads were assembled by StringTie (v1.3.3b). To gauge gene expression abundance we used FPKM (fragments per kilobase of transcript per million mapped reads), the prevailing approach for this purpose. The DESeq2 R package (v1.20.0) was employed to execute differential expression analysis, with subsequent adjustment of resulting *P*-values utilizing the Benjamini–Hochberg method to control the false discovery rate. Differentially expressed genes were designated based on the criteria of |log_2_(FoldChange)| ≥ 1 and *P*_adj_ ≤ 0.05.

### Proteomics/phosphoproteomics data analysis and bioinformatics

#### Protein sample lysis and digestion

Frozen plant tissue samples were pulverized using a tissue lyzer in liquid nitrogen, followed by extraction in a lysis buffer. Tris-saturated phenol (pH 8.0) in an equivalent volume was introduced, followed by vigorous vortexing for 5 min. Following centrifugation at 5000 *g* for 10 min, the upper phenol phase was carefully collected. Proteins were then precipitated overnight at −20°C using four volumes of ammonium sulfate-saturated methanol, followed by three thorough washes with ice-cold acetone. The desiccated protein samples were reconstituted in a urea solution (8 M urea, 50 mM Tris–HCl, pH 7.5) supplemented with phosphatase inhibitor (Sigma–Aldrich) and cOmplete EDTA-free protease inhibitor cocktail (Roche).

One hundred micrograms of protein underwent reduction using dithiothreitol at 56°C for 30 min, followed by alkylation using iodoacetamide for 15 min in darkness at 25°C. Subsequently, triethylammonium bicarbonate (TEAB) was added to dilute the protein sample to achieve a urea concentration of <2 M. After that, trypsin digestion was performed by incubation at 37°C with gentle vibration overnight. The peptide samples were acidified to pH 2 with formic acid, followed by centrifugation at 12 000 *g* for 10 min. The resulting supernatant was then subjected to desalting on a Strata-X SPE column.

#### Desalting

The Strata-X SPE column was conditioned with 1 ml methanol and 2 ml H_2_O [0.1% trifluoroacetic acid (TFA)], sequentially. Peptide solutions were applied to the cartridge and allowed to drip by gravity. Then the cartridges were rinsed three times with 1 ml 0.1% TFA. Peptides were eluted in 1 ml 80% acetonitrile (0.1% TFA) and vacuum-dried in a SpeedVac (Labconco, USA).

#### Phosphopeptide enrichment

The peptide mixtures underwent incubation with an IMAC microsphere suspension in a loading buffer comprising 50% acetonitrile and 0.5% acetic acid, accompanied by continuous vibration. To eliminate non-specifically adsorbed peptides, the IMAC microspheres were subjected to sequential washes, first with 50% acetonitrile containing 0.5% acetic acid, and then with 30% acetonitrile containing 0.1% TFA. The enriched phosphopeptides were eluted with vibration using 10% NH_4_OH buffer. Subsequently, the supernatant was lyophilized for further LC–MS/MS.

### LC–MS/MS analysis

The peptides were solubilized in H_2_O containing 0.1% formic acid and 2% acetonitrile. Peptide separation was achieved through a gradient of 0.1% formic acid in acetonitrile, starting at 2% and increasing to 22% in 16 min, increasing from 22% to 35% in 6 min, a steep ascent to 90% over 4 min, and remaining at 90% for the final 4 min. All steps were carried out at 450 nl/min using a nanoElute UPLC system (Bruker Daltonics).

After capillary ionization, the peptides were analyzed using a timsTOF Pro (Bruker Daltonics) mass spectrometer with an electrospray voltage of 1.70 kV. The TOF detector was employed for the analysis of both precursors and fragments, covering an MS range of 100–1700 *m*/*z*. After the initial mass spectrometry data acquisition, the timsTOF Pro operated in data-independent acquisition parallel accumulation serial fragmentation (DIA-PASEF) mode, featuring an MS/MS scan range of 400–1200 *m*/*z* and a fixed isolation window of 25 *m*/*z*.

### Protein/phosphoprotein identification and quantification

The resulting DIA data were analyzed with the Spectronaut search engine (v16.0). Mass spectra were queried against a concatenated database consisting of the *S. baicalensis* sequence database (containing 30 100 entries) and its corresponding reverse decoy database. The specific cleavage enzyme selected was trypsin/P, allowing for a maximum of two missed cleavages. The mass tolerance of precursor ions was adjusted to 20 and 5 ppm in the initial search and main search, respectively. Additionally, the mass tolerance for fragment ions was established as 0.02 Da. Cysteine residues underwent fixed carbamidomethylation, and variable modifications encompassed protein acetylation and methionine N-terminal oxidation. Phosphorylation modification (+79.97) on serine, threonine, and tyrosine was used as a variable modification in the phosphoproteomics. MSstats R was used to normalize protein intensity and FDR was adjusted to ≤1%.

### Motif analysis

Omics experiments focusing on large-scale modifications can identify thousands of protein post-translational modification sites, and it is crucial to comprehend the potential biological processes influenced by these modifications. Peptide sequences, comprising six amino acids both upstream and downstream of all the identified modification sites, underwent assessment using the MoMo software tool. A sequence is classified as a motif for the modified peptide segments when the number of characteristic sequence peptides is >20 and the *P*-value is <0.000001. Using the MoMo outcomes, the degree score (DS) of amino acids reflecting the frequency shift surrounding the modification sites was depicted through a sequence logo map. The algorithm for computing DS is expressed as follows: DS = −log_10_(*P* value) × sign (diff. percent).

### Functional analysis

GO enrichment analysis was performed and categorized into three broad classifications. The Pfam and KEGG databases were utilized for functional enrichment analysis. The significance of enrichment of phosphoproteins was calculated using Fisher’s exact test, with the identified proteins as the background and *P* ≤ 0.05 as a threshold. Protein–protein interaction (PPI) analysis was conducted utilizing the STRING database (https://string-db.org/cgi/input.pl).

### Western blotting analysis

Total proteins were extracted with buffer (50 mmol/l Tris–HCl, pH 7.5, 150 mmol/l NaCl, 5 mmol/l β-mercaptoethanol, 1% Triton X-100 v/v, 1 mmol/l PMSF) and centrifuged to collect supernatants at 12 000 g for 10 min at 4°C. Proteins were detected using rabbit anti-CHS antibodies (P42500R2, 1:500, Abmart, China), followed by secondary antibodies, IRDye^®^ 800CW Goat anti-Mouse (926-32 210, LI-COR, USA). Anti-tubulin antibodies (M30109, 1:1000, Abmart) served as reference control. Signal detection was performed with an Odyssey CLx Imaging System (LI-COR, USA).

### Parallel reaction monitoring experimental procedures

The enriched tryptic peptides were introduced into the NSI source using a Q Exactive™ Plus instrument (Thermo). The ensuing MS data were subsequently processed via Skyline (v3.6). Regarding peptide settings, the specified enzyme was trypsin (KR/P), with a maximum allowed missed cleavage set at 2. Peptide lengths were limited to the range of 8–25 amino acids. Variable modifications included carbamidomethyl on cysteine (Cys) and oxidation on methionine (Met), with a maximum of three variable modifications allowed. In the context of transition settings, precursor charges were constrained to 2 and 3, with ion charges limited to 1 and 2. Mass tolerance of product ions was 0.02 Da.

### Data analysis

Sample similarity scores were calculated based on Pearson correlations across all proteins/transcripts identified in the complete set of samples. *P* values were adjusted using the Benjamini–Hochberg method to control *P*_adj_ at a cutoff level of 0.05, with the criterion of |log_2_(FoldChange)| ≥ 1 for differential transcripts. For differential proteins, the threshold was set at |log_2_(FoldChange)| ≥ 1 and *P* ≤ 0.05.

## Acknowledgements

This work was supported by the National Natural Science Foundation of China (82104328, 81973291, 82122066), the National Key Research and Development Program of Ministry of China (No. 2022YFC2704603), and the Shanghai Sailing Program (No. 20YF1458900). We thank Dr Fanwang Meng and Prof. Jingbo Zhang for helpful comments on the manuscript.

## Author contributions

D.D.G. and X.F.C. initiated the program, coordinated the project, and wrote the manuscript. D.D.G., F.F., Z.W.X., and D.Y.L. prepared and analyzed the samples. D.D.G., Z.Y. Z., Z.W., Q.C.Z., W.X., and X.L.J. performed the data analysis. X.F.C. and T.H. provided critical comments.

## Data availability statement

The transcriptome sequencing for endometrium samples have been deposited with the China National Center for Bioinformation under reference number PRJCA016351(https://ngdc.cncb.ac.cn/search/?dbId=bioproject&q=PRJCA016351). The MS proteome and phosphoproteome data were submitted to iProX (integrated protein resource) (https://www.iprox.cn/page/home.html) under the reference numbers PXD041602 and PXD041603).

## Conflict of interest

The authors declare no competing commercial interests.

## Supplementary information

Supplementary data is available at *Horticulture Research* online.

## Supplementary Material

Web_Material_uhad258
