# Supplementary material for: Multi-omics landscape to decrypt the distinct flavonoid biosynthesis of Scutellaria baicalensis across multiple tissues
Source: Hortic Res. 2023 Nov 13;11(1):uhad258. doi: 10.1093/hr/uhad258 (PMC10828779; doi:10.1093/hr/uhad258)
Supplement: Web_Material_uhad258 [file web_material_uhad258.zip › Figure S2.pdf]

```
UGAT1       ....................................MNTMAVLAKFISKNHPSVPIIIISNAPESAAAS.VAAIPSISYHRLELPEIPPDMTTDRVELFF   63
SbUGAT4     ..........................................................................................MTTDRVELFF   10
SbUGAT1.1   MLINTESQTHNKQKEKVGCMEKSMEGSIVMYGVEQHLNTVSLLAKFIGTKQPSVPIIIISSAEESAVAASIAAVPSISYHRLELPAPPPNLTKDPVELLF  100
SbUGAT1.2   ...................MEKSMEGSIVMYVVEQHMNTMSLLAKFIGTKQPSVPIIIISSAEESVVAASIAAVPSISYHRLELPAPPPNLTKDPVELLF   81
SbUGAT2     .......................MEDIIAIYCVESHLNTVSVLAKFISKHQPSVPIVIICAATKSDVAS.IAAVPSVTYHLLEAPPLPPNFTKNPVELFF   76
UGAT6       ......................MEEDTIVIYSAESHMNTMSLLAKFISKHHPKIPIVIICTADADSAAA.VSGVPSITYRRLETEALPPDITDNCVELMF   77
SbUGAT3     ......................MEEDTIVIYSAEGHMNTMSLLAKFISKHHPKIPIVIICTADADSAAA.VSGVPSITYRRLETEALPPDITDNCVELMF   77
UGAT2       .......................MADTMVLYSTPEHLNSMLVLARFIGKHHPSVPVIIISYAAECA....VAAGPSVTYHRLEIPTLPADLTKDHLELYF   73
SbUGAT5     .......................MADTMVLYSTPEHLNSMLVLARFIGKHHPSVPVIIISYAAECA....VAAGPSVTYHRLEIPTLPADLTKDHLELYF   73
UGAT7       .......................MEDTIVLYASPEHLNSMLVLAKFISKHHPSVPIIIISTASESAATS.IATVPSVTYRRLEDPTLPPNMTKDPVELFF   76
SbUGAT6     .......................MEDTIVLYASPEHLNSMLVLAKFISKHHPSVPIIIISTASESAATS.IATVPSVTYRRLEDPTLPPNMTKDPVELFF   76

UGAT1       ELPRLSNPNLLTALQQISQKTRIRAVIIDFFCNAAFEVPTSLNIPTYYYFSAGTPTAILTLYFETIDETIPVDLQDLNDYVDFPGLPPIHCLDIPVALLT  163
SbUGAT4     ELPRLSNPNLLTALQQISQKTRIRAVILDFFCNAAFEVPTSLNIPTYYYFSAGTPTAILTLYFETIDETIPVDLQDLNDYVDIPGLPPIHCLDIPVALLT  110
SbUGAT1.1   ELPRLNNPNVRDALQQISQKARIRAFVVDLFCHTACEVSQSLNVPTFLYLSSGAAFAILCLCFQGIHESMSVDTGELNDFFELPGLPPIPSLDLPVFGCS  200
SbUGAT1.2   ELPRLNNPNVRDALQQISQKARIRAFVVDFLCHTACEVSQSLNVPTFLYLSSGAAFTIFCLCFQGIHESMSVDTGELNDFFELPGLPTISSMDLPVFGCS  181
SbUGAT2     EIPRLNNPKLHDSLLEISEKSRIRTLVLDVFCNSAFEVCESLKIPTYYYFSAGSAMVVLFMYFATLDDTMRVDLGELNDYVEMPGLPPIYSLDFPKDVYS  176
UGAT6       EIPRLNNANLREALHEISQKARIRAFIIDFFCNSGFEVSESMEIPTYHYFSAGTAMAIYSLCYAEIHETITVDLGEFKDFIEFPGLPLIYSLDFPRDALL  177
SbUGAT3     EIPRLNNANLREALHEISQKARIRAFIIDFFCNSGFEVSESMEIPTYHYFSAGTAVAIYSLCYAEIHETITVDLGEFKDFIEFPGLPLIYSLDFPRDALL  177
UGAT2       EIPRVMNPSLHHALQEISRKATIKAFVIDFFCNAAFEVSTGLGIPTYFYVSTGGFGLSAFLYFPTLDETIAGDIGQLDDFLEIPGCPLVHSSDFPKGMHF  173
SbUGAT5     EIPRVMNPSLHHALQEISRKATIKAFVIDFFCNAAFEVSTGLGIPTYFYVSTGGFGLSAFLYFPTLDETIARDIGELDDFLEIPGCPLVHSSDFPKGMHF  173
UGAT7       ELPRLNNPNLRDCLAEISEKTRIGAFVMDYFCNAAFEVCMSLNIATYYYVSSGAFGLCIILHFPTIDGTIGPDIGDFNDFLEFPGCPPLHSSDIPKGMHF  176
SbUGAT6     ELPRLNNPNLRDCLAEISEKTRIGAFVMDYFCNAAFEVCMSLNIATYYYVSSGAFGLCIILHFPTIDGTIGPDIGDFNDFLEFPGCPPLHSSDIPMGMHF  176

UGAT1       RKSLVYKSSVDISKNLRRSAGILVNGFDALEFRAKEAIVNGLCISKGPTPPVYFIGPLVGDVDTKAGSEDHECLRWLDTQPSKSVVFLCFGRRGVFSAKQ  263
SbUGAT4     RKSLVYKSSVDISKNLRRSAGILVNGFDALEFRAKEAIVNGLCISKGPTPPVYFIGPLVGDVDTKAGSEEHECLRWLDTQPSKSVVFLCFGRRGVFSAKQ  210
SbUGAT1.1   RQGLFYKHMVGVSENLQKSRGIIVNAFDALEYRAKEAISNGLCLQNGPTPPVYFMGPLISDIQG..GGEEHECMRWLDKQPSKSVIFLCFGRRCVFSAEQ  298
SbUGAT1.2   RQGLFYKHLVGVSENLQKSRGIIVNAFDALEYRAKEAISNGLCLQNGPTPPVYFMGPLISDIQG..GGEEHECMRWLDKQPSKSVIFLCFGRRCVFSAEQ  279
SbUGAT2     RGSLVYKHVLEVSKNLQKSRGVMVNAFDALEMRAKEAISNGLCLPNGATPPVYYIGPLVEDVK...AEEEHECLSWLDKQPSKSVVFLCFGRRGMFSAEQ  273
UGAT6       RQSIFNKHMVCLSKYMRKSRGFILNAFDALDYRGKEAISNGLCLPNEPTPPVYFMGPLVGDVE...GGAKHECLSWLDKQPSKSVVFLCFGRRGVFSAEQ  274
SbUGAT3     RQSIFNKHIVCLSKYMRKSRGFIVNAFDALDYRAKEAISNGLCLPNEPTPPVYFMGPLVGDVE...GGAKHECLSWLDKQPSKSVVFLCFGRRGVFSAEQ  274
UGAT2       RQSNIYKHFLSTGKNLRRAAGIVANTFDALEYRSKEALANGLCVPGGPTPPVYLVGPLVAGGGSKTGGE..ECLKWLDKQPSKSVIFLCFGRRGLFSGEQ  271
SbUGAT5     RQSNIYKHFLSTGKNLRRAAGIVANTFDALEYRSKEALANGLCVPGGPTPPVYLVGPLVAGGGSKTGGE..ECLKWLDKQPSKSVIFLCFGRRGLFSGEQ  271
UGAT7       RQSNTYKHFKSAGINMARSAGIVVNSFDALEKRAKEALDKGLSIPNAPTPPVYFIGPLVAEPDAKNSGEEHECLRWLEKQPSRSVVFLCFGRRGLFSGEQ  276
SbUGAT6     RQSNTYKHFKSAGINMARSAGIVVNSFDALEKRAKEALDKGLSIPNAPTPPVYFIGPLVAEPDAKNSGEEHECLRWLEKQPSRSVVFLCFGRRGLFSGEQ  276

                                                                                    PSPG motif
                                                                    --------------------------------
UGAT1       LKETAAALENSGHRFLWSVRNPPELKKATGSDEPDLDELLPEGFLERTKDRGFVIKSWAPQKEVLAHDSVGGFVTHCGRSSLSEGVWFGVPMIGWPVDAE  363
SbUGAT4     LKETAAALENSGHRFLWSVRNPPELKKATGSDEPDLDELLPEGFLERTKDRGFVIKSWAPQKEVLAHDSVGGFVTHCGRSSVSEGVWFGVPMIGWPVDAE  310
SbUGAT1.1   LKETAMGLEKSGHRFLWAVRNPVD........EADLNEVLPKGFVERTKDRGFVIESWGPQKEVLSHDSVGGFVTHCGRSSLSEALWFGVPMIGWPVDAE  390
SbUGAT1.2   LKETAMGLEKSGHRFLWAVRNPVD........EADLNEVLPKGFVERTKDRGFVIESWAPQKEVLSHNSVGGFVTHCGRSSLSEALWFGVPMIGWPVDAD  371
SbUGAT2     LKETAVGLEKSGHRFLWSLRTPPG........TDEAAELLPEGFVERTKDRGFVIKSWAPQKEVLSHDSVGGFVTHCGRSSVSEALWFGVPMIGWPVYAE  365
UGAT6       LKETAIGLEKSGHGFLWAVRNPMDG......GEADLDAALPEGFMERTKERGLVVKSWAPQREVLSHDSVGGFVTHCGRSSIFEGLWFGVPMIGWPMDAE  368
SbUGAT3     LKETAIGLEKSGHRFLWAVRSPMDG......GEADLDAALPEGFMERTKERGLVVKSWAPQREVLSHDSVGGFVTHCGRSSIFEGLWFGVPMIGWPMDAE  368
UGAT2       LKEMAVGLENSGHRFLWSVRNPPGKQ....SKERDLDDLLPEGFLERTKDRGFVVTSWAPQTAVLSHESVAGFVTHCGRSSFLEAVSFGVPMIAWPLYAE  367
SbUGAT5     LKEMAVGLENSGHRFLWSVRNPPGKQ....SKERDLDDLLPEGFLERTKDRGFVVTSWAPQTAVLSHESVAGFVTHCGRSSFLEAVSFGVPMIAWPLYAE  367
UGAT7       LKAMAVGLENSGHRFLWSVRSPPEKQD...ATAAELDELLPEGFLERTRDRGFVVKSWAPQTAVLSHGAVAGFVTHCGRSSILEAVSFGVPMIAWPLYAE  373
SbUGAT6     LKAMAVGLENSGHRFLWSVRSPPEKQD...ATAAELDELLPEGFLERTRDRGFVVKSWAPQTAVLSHGGVAGFVTHCGRSSILEAVSFGVPMIAWPLYAE  373

UGAT1       QKLNRAVAVDDLQVALPLEEEAGGFVTAAELEKRVRELMET..KAGKAVRQRVTELKFSARAAVAENGSSLNDLKKFLLATRD                   444
SbUGAT4     QRLNRAVMVDDLQVALPLEEEAGGFVTAAELEKRVRELMET..KAGKAVRQRVTELKFSARAAVAENGSSLNDLKKFLLATRD                   391
SbUGAT1.1   QRLNRTVMVEEMQVALPLEEEAHGFVTATELEKRVRELMDS..HKVREMRRRVSEMKSSAMAAMAENGSSSIDLHNFLLFTAH                   471
SbUGAT1.2   QRLNRTVMVEEMQVALPLEEEAYGFVTATELEKRVRELMDS..HKGREMRRRVSEMKSSARAAMAENGSSSIDLHNFLLFTAH                   452
SbUGAT2     QRMNRVVMVEEMRVALPLEEVAPG................................GGSVAAAR..................                   397
UGAT6       QRMNRTVVVEDMRVVLPLEEAADEFVTAAELEKRVRELMDSESKTGKEVRRRVGEMKSSARKAMAENGSSVVDLHKFLLSATL                   451
SbUGAT3     QRMNRTVVVEDMRVVLPLEEAADEFVTAAELEKRVRELMDSESKTGKEVRRRVGEMKSSARKAMAENGSSVVDLHKFLLSATL                   451
UGAT2       QRMNRAFMVEEMQLALPVEAAAEGLVAAAELEKRVKELMES..KAGRAVRQRITEMKHAACAAVRPKGSALLALEKFITAVTR                   448
SbUGAT5     QRMNRAFMVEEMQLALPVEAAAEGLVAAAELEKRVKELMES..KAGRAVRQRITEMKHAACAAVRPKGSALLALEKFITAVTR                   448
UGAT7       QRVNRVFMVKEMGVALPLEEAADGFVAAAELEKRVKELMES..KAGEEVRERVTEMKKAAMAAMREKGSSLLALQKLITAVV.                   453
SbUGAT6     QRVNRVFMVKEMGVALPLEEAADGFVAAAELEKRVKELMES..KAGEEVRERVTEMKKAAMAAMREKGSSLLALQKLITAVV.                   453
```
