# Supplementary material for: Multi-omics landscape to decrypt the distinct flavonoid biosynthesis of Scutellaria baicalensis across multiple tissues
Source: Hortic Res. 2023 Nov 13;11(1):uhad258. doi: 10.1093/hr/uhad258 (PMC10828779; doi:10.1093/hr/uhad258)
Supplement: Web_Material_uhad258 [file web_material_uhad258.zip › Figure S5.pdf]

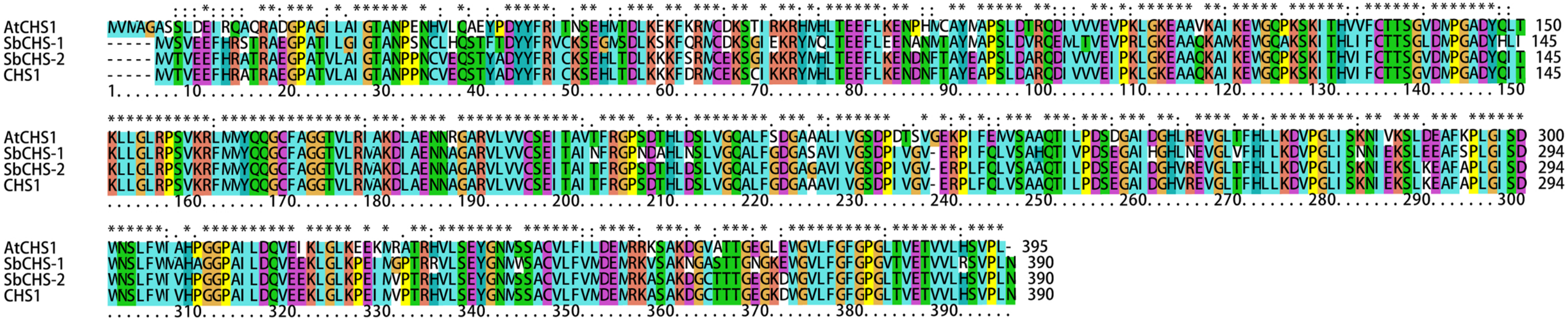
AtCHS1 MVMAGASSLDEIRQAQRADGPAGILAIGTANPENHVLQAEYPDYYFRITNSEHMTDLKEKFKRMCDKSTIRKRHMHLTEEFLKENPHMCAYMAPSLDTRQDIVVVEVPKLGKEAAVKAIKEWGQPKSKITHVVFCTTSGVDMPGADYQLT 150
SbCHS-1 -----MVSVEEFHRSTRAEGPATILGIGTANPSNCLHQSTFTDYYFRVCKSEGMSDLKSKFQRMCDKSGIEKRYMQLTEEFLEENANMTAYMAPSLDVRQEMLTVEVPRLGKEAAQKAMKEWGQAKSKITHLIFCTTSGLDMPGADYHLI 145
SbCHS-2 -----MVTVEEFHRATRAEGPATVLAIGTANPPNCVEQSTYADYYFRICKSEHLTDLKKKFSRMCEKSGIKKRYMHLTEEFLKENDNFTAYEAPSLDARQDIVVVEIPKLGKEAAQKAIKEWGQPKSKITHVIFCTTSGVDMPGADYQIT 145
CHS1 -----MVTVEEFHRATRAEGPATVLAIGTANPPNCVEQSTYADYYFRICKSEHLTDLKKKFDRMCEKSCIKKRYMHLTEEFLKENDNFTAYEAPSLDARQDIVVVEIPKLGKEAAQKAIKEWGQPKSKITHVIFCTTSGVDMPGADYQIT 145
AtCHS1 KLLGLRPSVKRLMMYQQGCFAGGTVLRIAKDLAENNRGARVLVVCSEITAVTFRGPSDTHLDSLVGQALFSDGAAALIVGSDPDTSVGEKPIFEMVSAAQTILPDSDGAIDGHLREVGLTFHLLKDVPGLISKNIVKSLDEAFKPLGISD 300
SbCHS-1 KLLGLRPSVKRFMMYQQGCFAGGTVLRMAKDIAENNAGARVLVVCSEITAINFRGPNDAHLNSLVGQALFGDGASAVIVGSDPIVGV-ERPIFQLVSAHQTIVPDSEGAIHGHLNEVGLVFHILKDVPGLISNNIEKSLEEAFSPLGISD 294
SbCHS-2 KLLGLRPSVKRFMMYQQGCFAGGTVLRMAKDLAENNAGARVLVVCSEITAITFRGPSDTHLDSLVGQALFGDGAGAVIVGSDPIVGV-ERPLFQLVSAAQTILPDSEGAIDGHVREVGLTFHLLKDVPGLISKNIEKSLKEAFAPLGISD 294
CHS1 KLLGLRPSVKRFMMYQQGCFAGGTVLRMAKDLAENNAGARVLVVCSEITAITFRGPSDTHLDSLVGQALFGDGAAAVIVGSDPIVGV-ERPLFQLVSAAQTILPDSEGAIDGHVREVGLTFHLLKDVPGLISKNIEKSLKEAFAPLGISD 294
AtCHS1 WNSLFWIAHPGGPAILDQVEIKLGLKEEKMRATRHVLSEYGNMSSACVLFILDEMRRKSAKDGVATTGEGLEWGVLFGFGPGLTVETVVLHSVPL- 395
SbCHS-1 WNSLFWVAHAGGPAILDQVEEKLGLKPEIMGPTRRVLSEYGNMWSACVLFVMDEMRKVSAKNGASTTGNGKEWGVLFGFGPGVTVETVVLRSVPLN 390
SbCHS-2 WNSLFWIVHPGGPAILDQVEEKLGLKPEIMVPTRHVLSEYGNMSSACVLFVMDEMRKASAKDGCTTTGEGKDWGVLFGFGPGLTVETVVLHSVPLN 390
CHS1 WNSLFWIVHPGGPAILDQVEEKLGLKPEIMVPTRHVLSEYGNMSSACVLFVMDEMRKASAKDGCTTTGEGKDWGVLFGFGPGLTVETVVLHSVPLN 390
