# Supplementary figures and images for: Multi-omics landscape to decrypt the distinct flavonoid biosynthesis of Scutellaria baicalensis across multiple tissues
Source: Hortic Res. 2023 Nov 13;11(1):uhad258. doi: 10.1093/hr/uhad258 (PMC10828779; doi:10.1093/hr/uhad258)

A

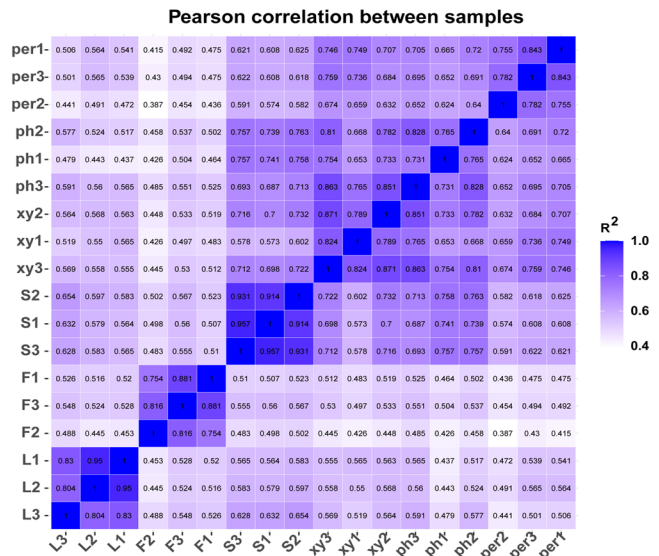

B

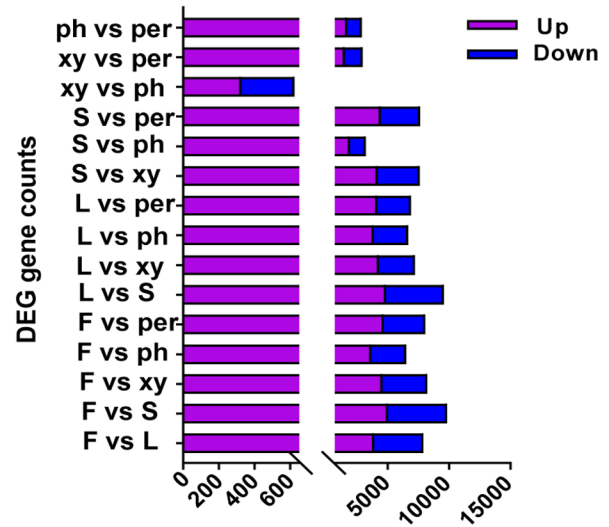

C

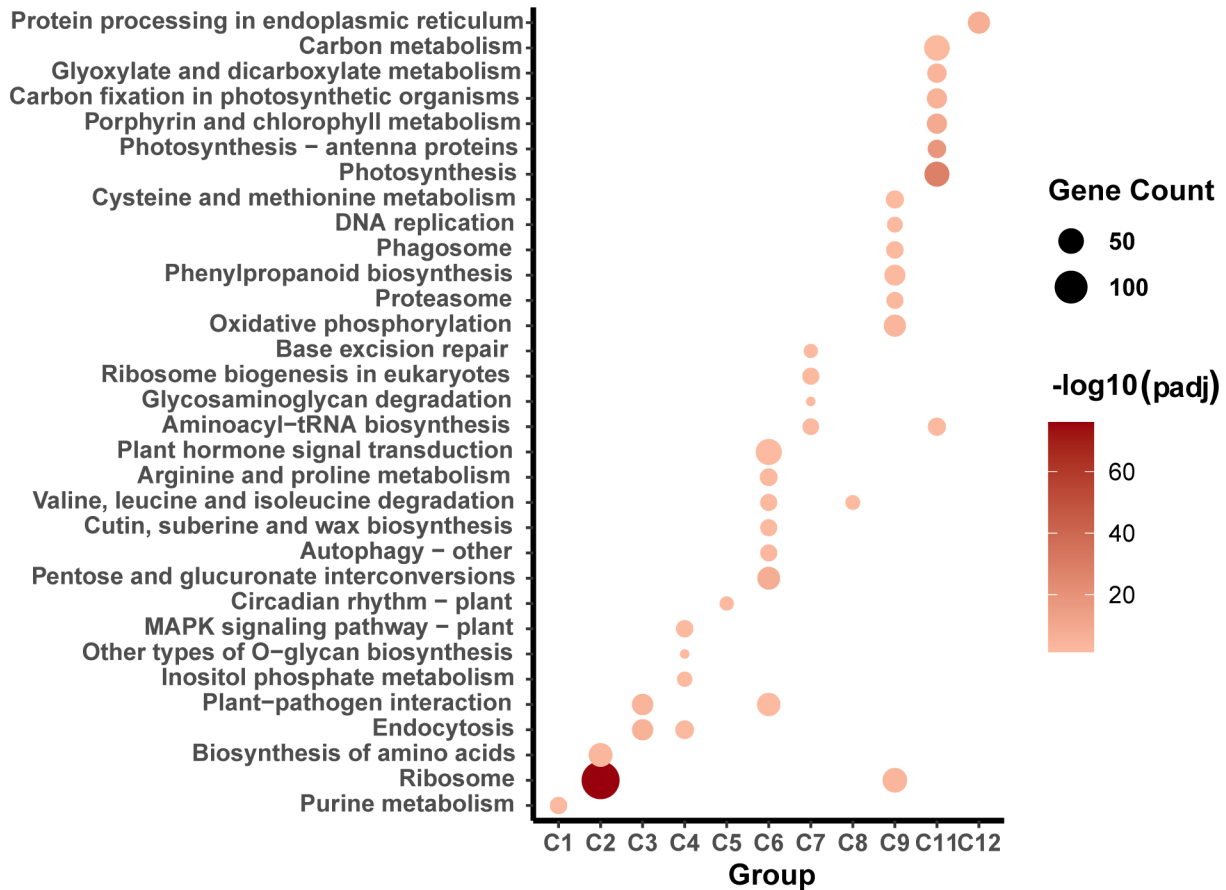

Supplement: Web_Material_uhad258 [file web_material_uhad258.zip › Figure S1.pdf]

A

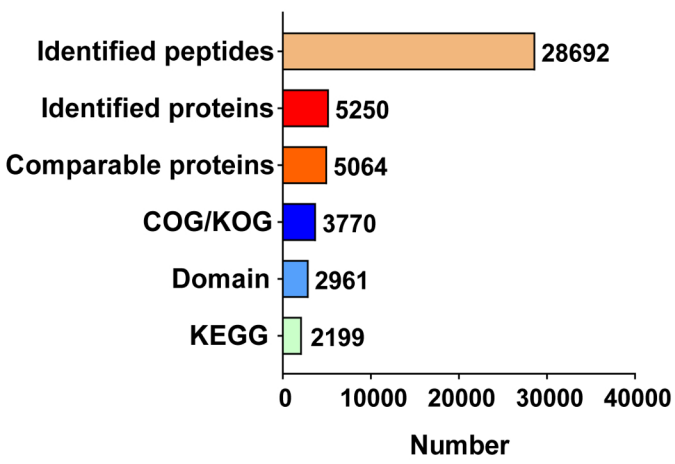

B

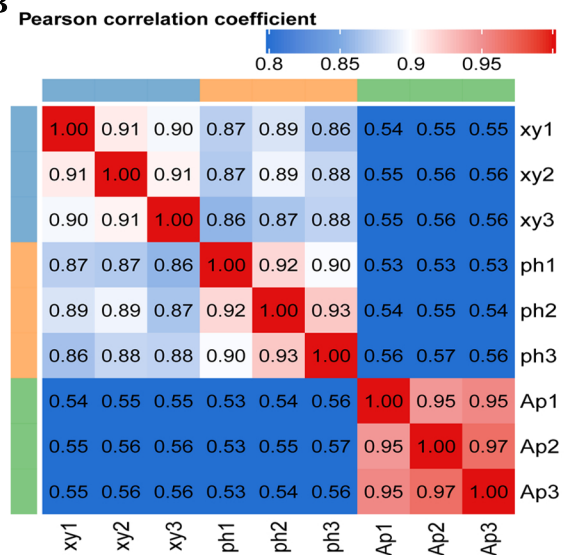

E

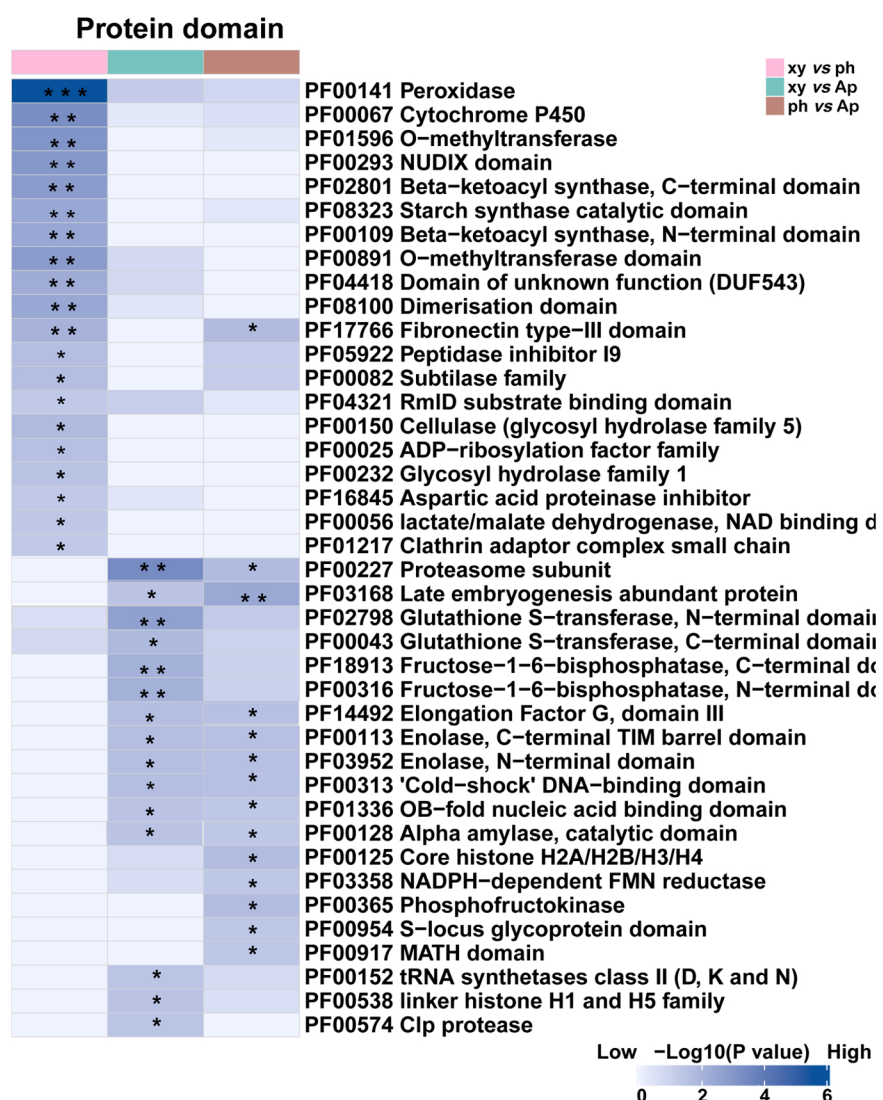

C

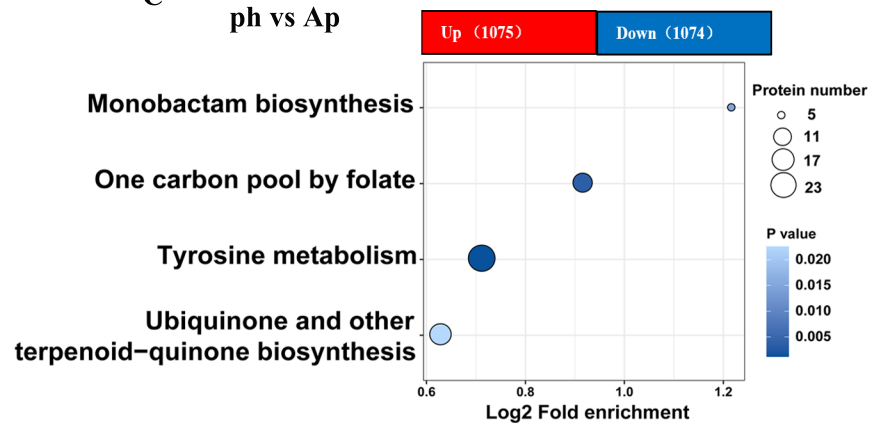

D

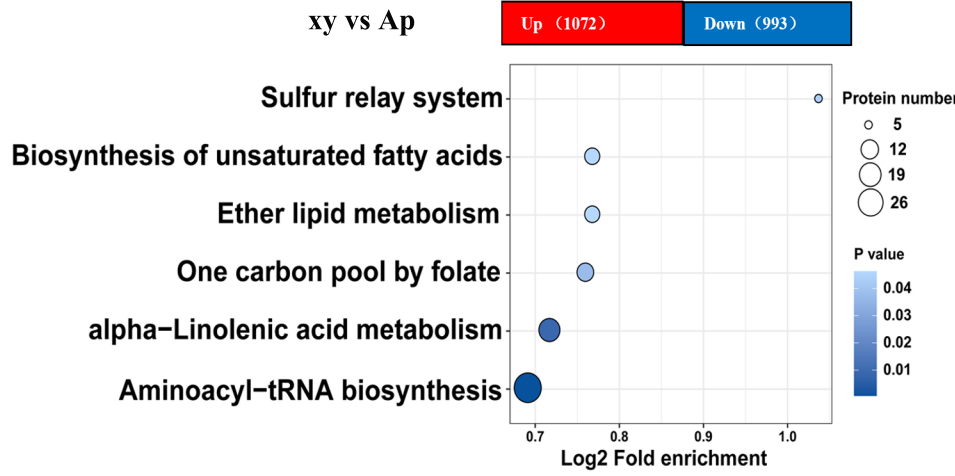

Supplement: Web_Material_uhad258 [file web_material_uhad258.zip › Figure S3.pdf]

A

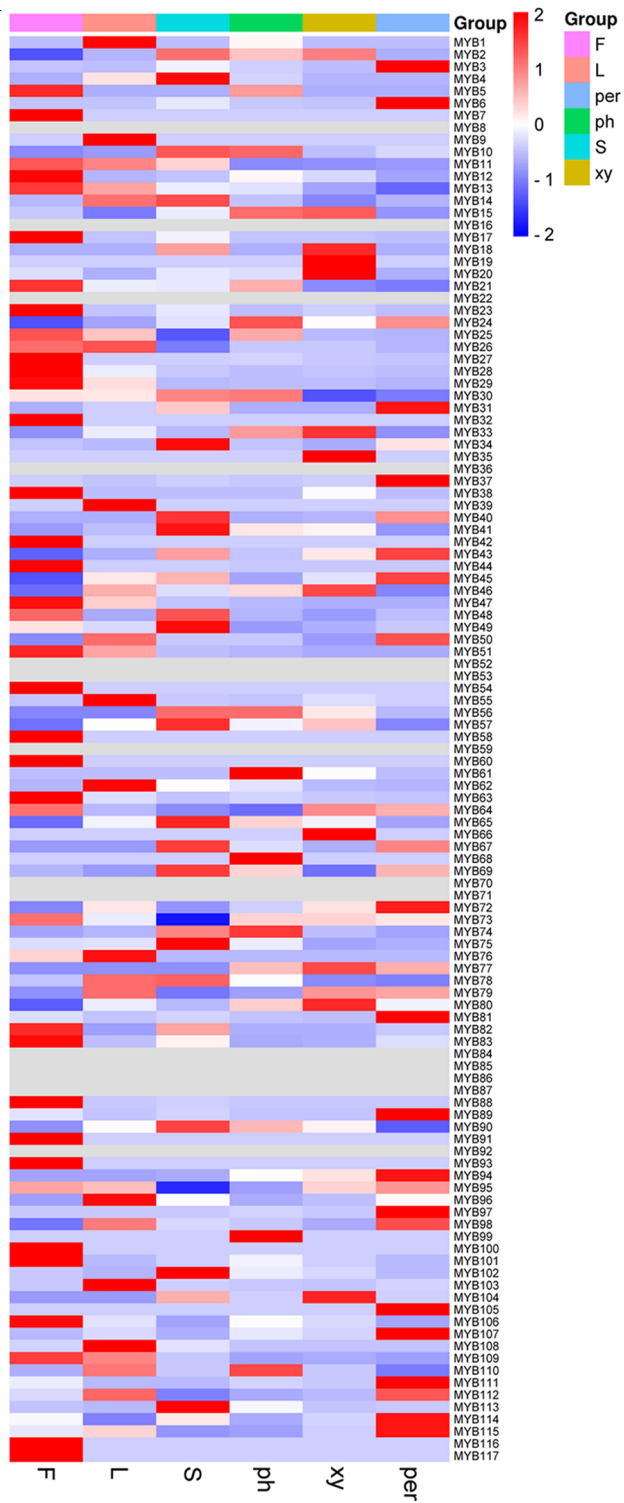

B

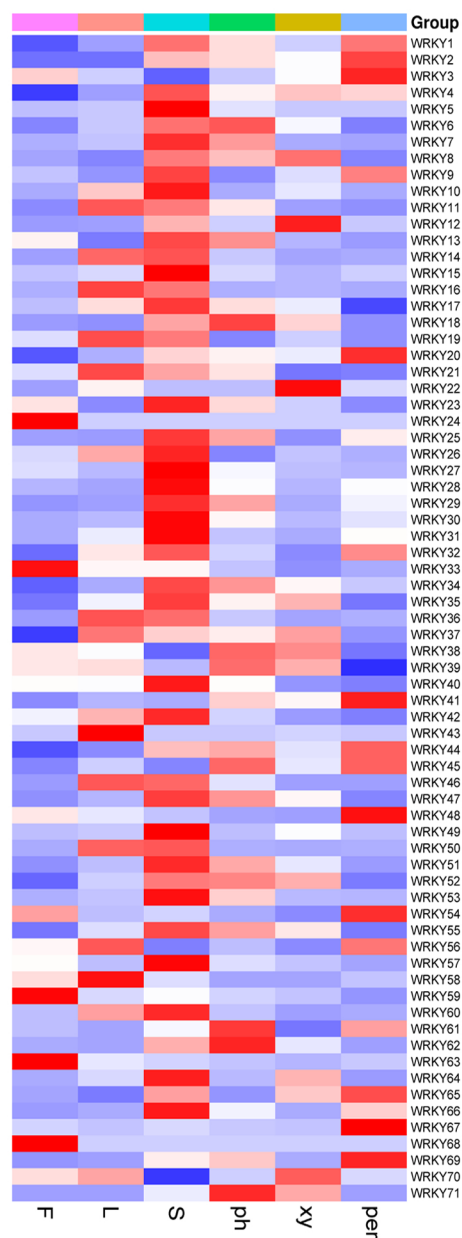

Supplement: Web_Material_uhad258 [file web_material_uhad258.zip › Figure S6.pdf]

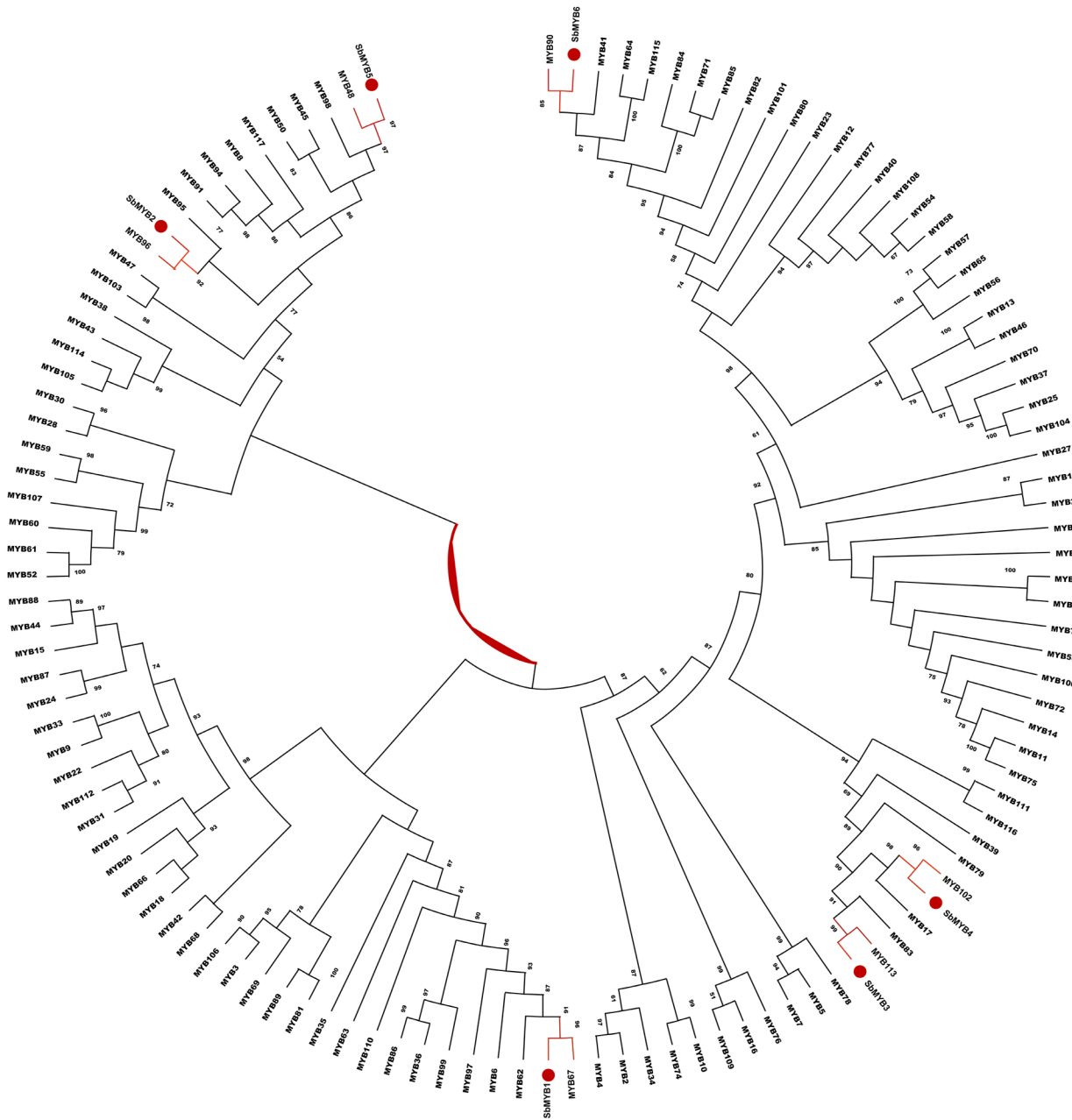

Supplement: Web_Material_uhad258 [file web_material_uhad258.zip › Figure S7.pdf]
