# Supplementary material for: Multi-omics landscape to decrypt the distinct flavonoid biosynthesis of Scutellaria baicalensis across multiple tissues
Source: Hortic Res. 2023 Nov 13;11(1):uhad258. doi: 10.1093/hr/uhad258 (PMC10828779; doi:10.1093/hr/uhad258)
Supplement: Web_Material_uhad258 [file Figure S4.pdf › Figure S4.pdf]

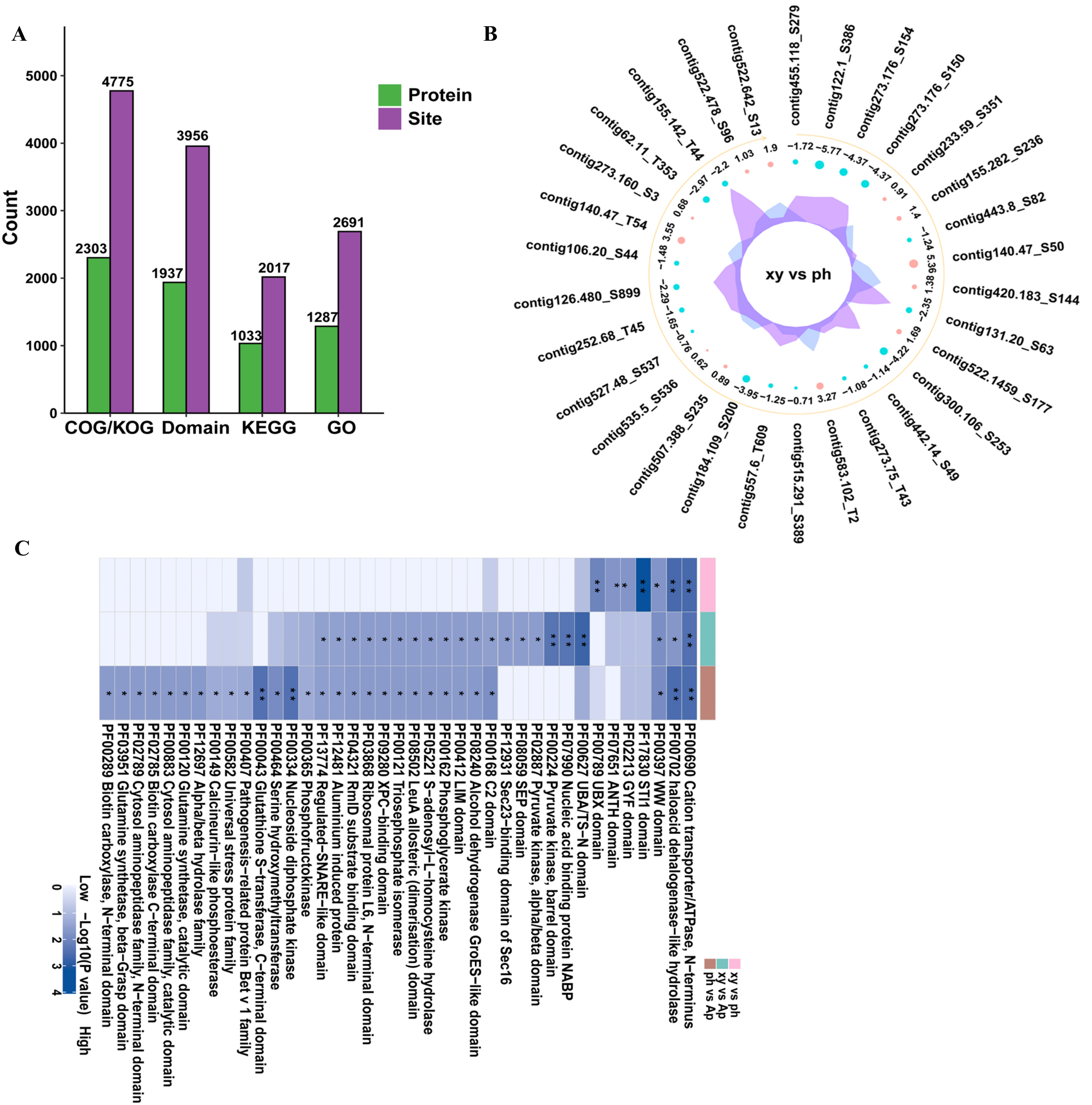
A
Count
Protein
Site
COG/KOG
Domain
KEGG
GO
2303
4775
1937
3956
1033
2017
1287
2691
B
xy vs ph
C
xy vs ph
xy vs Ap
ph vs Ap
Low −Log10(P value) High
PF00690 Cation transporter/ATPase, N−terminus
PF00702 haloacid dehalogenase−like hydrolase
PF00397 WW domain
PF17830 STI1 domain
PF02213 GYF domain
PF07651 ANTH domain
PF00789 UBX domain
PF00627 UBA/TS−N domain
PF07990 Nucleic acid binding protein NABP
PF00224 Pyruvate kinase, barrel domain
PF02887 Pyruvate kinase, alpha/beta domain
PF08059 SEP domain
PF12931 Sec23−binding domain of Sec16
PF00168 C2 domain
PF08240 Alcohol dehydrogenase GroES−like domain
PF00412 LIM domain
PF00162 Phosphoglycerate kinase
PF05221 S−adenosyl−L−homocysteine hydrolase
PF08502 LeuA allosteric (dimerisation) domain
PF00121 Triosephosphate isomerase
PF09280 XPC−binding domain
PF03868 Ribosomal protein L6, N−terminal domain
PF04321 RmlD substrate binding domain
PF12481 Aluminium induced protein
PF13774 Regulated−SNARE−like domain
PF00365 Phosphofructokinase
PF00334 Nucleoside diphosphate kinase
PF00464 Serine hydroxymethyltransferase
PF00043 Glutathione S−transferase, C−terminal domain
PF00407 Pathogenesis−related protein Bet v 1 family
PF00582 Universal stress protein family
PF00149 Calcineurin−like phosphoesterase
PF12697 Alpha/beta hydrolase family
PF00120 Glutamine synthetase, catalytic domain
PF00883 Cytosol aminopeptidase family, catalytic domain
PF02785 Biotin carboxylase C−terminal domain
PF02789 Cytosol aminopeptidase family, N−terminal domain
PF03951 Glutamine synthetase, beta−Grasp domain
PF00289 Biotin carboxylase, N−terminal domain
